# Allelopathic Polyketides from an Endolichenic Fungus *Myxotrichum* SP. by Using OSMAC Strategy

**DOI:** 10.1038/srep19350

**Published:** 2016-02-03

**Authors:** Chao Yuan, Yu-Hua Guo, Hai-Ying Wang, Xiao-Jun Ma, Tao Jiang, Jun-Ling Zhao, Zhong-Mei Zou, Gang Ding

**Affiliations:** 1 Institute of Medicinal Plant Development Yunnan Branch, Chinese Academy of Medical Sciences and Peking Union Medical College, Jinghong, P.R. China; 2 Institute of Medicinal Plant Development, Chinese Academy of Medical Sciences and Peking Union Medical College, Beijing, P.R. China; 3 College of Life Sciences, Shandong Normal University, No. 88 East Wenhua Road, Jinan, P.R. China

## Abstract

Three new polyketides myxotritones **A**-**C** (**2**–**4**), together with a new natural product 7,8-dihydro-7*R*,8*S*-dihydroxy-3,7-dimethyl-2-benzopyran-6-one (**1**) were obtained from the endolichenic fungus *Myxotrichum* sp. by using OMSAC (One Strain, Many Compounds) method. The planar structures of these new compounds were determined by NMR experiment and HRESIMS data, and the absolute configuration of **1** was established by X-ray diffraction, and the stereochemistry of the new compounds **2**-**4** were determined by same biosynthesis origin, and similar CD spectra with **1**. Allelopathic test showed that compound **4** significantly retarded root elongation of *Arabidopsis thaliana* seed, indicating that this fungus might contribute to the defense of its host lichen. From the view of biosynthetic pathway, all four compounds **1**-**4** might be originated from Non-Reduced Polyketide synthase (NR-PKS).

Lichens are combinations of a fungus (the mycobiont) and an algal partner (the photobiont or phycobiont). In addition to fungal mycobionts, some nonobligate fungi, such as endolichenic fungi, are also found to live asymptomatically in the bodies (thalli) of lichens^1^. Although endolichenic fungi inhabit the lichen thalli similarly to endophytes living in the intercellular spaces of healthy plant tissues, the chemistry of this class of fungi remained largely unexplored^2^.

Analysis of a great number genome sequence from different microbes revealed that many secondary metabolite biosynthetic gene clusters are silent under common cultivation conditions, and their metabolic potentials were underestimated. To activate the cryptic gene cluster to express, different methods were innovated^3^. One easiest way is to vary the culture media to induce different cryptic gene cluster to express and then obtain new/novel secondary metabolites. This approach was termed as “One Strain, Many Compounds” first suggested by Germany natural product chemist Prof. A Zeek^4^. In our previous report, a series of citromycetin and fulvic acid with unique skeletons have been obtained from the PDB culture of endolichenic fungus *Myxotrichum* sp^5^ (Fig. 1). To dig the metabolic potential of endolichenic fungi *Myxotrichum* sp., rice culture was used to activate the potentially silent gene clusters, from which three new polyketides myxotritones A-C (**2**–**4**), together with a new natural product 7, 8-dihydro-7*R*, 8*S*-dihydroxy-3, 7-dimethyl-2-benzopyran-6-one (**1**) were obtained. In this report, the structural elucidation, biological evaluation, and possible biosynthetic pathway were present.

## Results and Discussion

**Figure 2:**
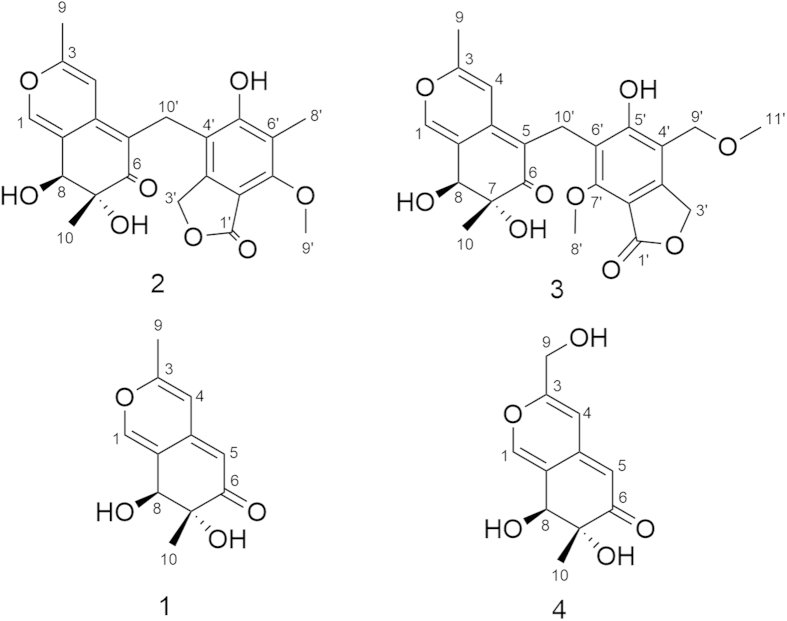
Structures of compounds (1–4).

**Figure 3:**
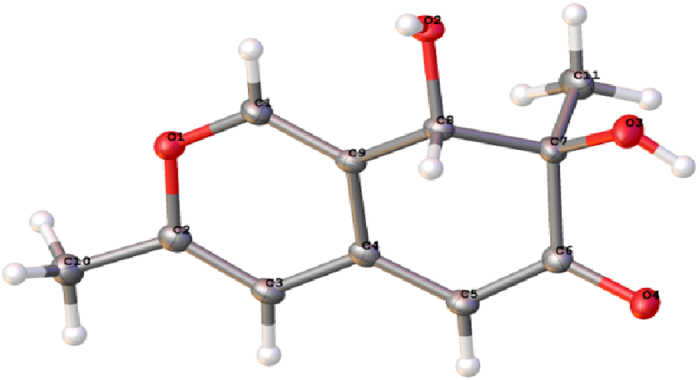
X-ray crystallographic structure of (1).

The known compound **1** is identified to be as 7, 8-dihydro-7*R*, 8*S*-dihydroxy-3, 7-dimethyl-2- benzopyran-6-one based on the NMR, MS data and optical rotation [α]_D_^22^ = +393.0 (*c* = 0.15, MeOH), which was known as a synthetic compound but never isolated from a natural specimen^6^,^7^ (Fig. 2). Fortunately, a suitable crystal was obtained for X-ray diffraction (in MeOH) (Fig. S5). The planar structure and absolute configuration of **1** were confirmed by single-crystal X-ray diffraction analysis with Cu Kα radiation (Fig. 3, CCDC 1419081). The CD spectrum of **1** showed the positive (362 nm, 311 nm and 225 nm) and negative (247 nm) cotton effects (Fig. S4) similar with those of known azaphilones clearly showed (*R*)-configuration of chiral center at C-7^8^,^9^,^10^. Snatzke’ rule was also used to determine the diol of C-7 and C-8^11^,^12^,^13^. The positive cotton effect at 327 nm observed *in situ* dimolybdenum CD spectra permitted the assignment of absolute configuration as 7*R*, 8*S* (Fig. S4).

**Figure 4:**
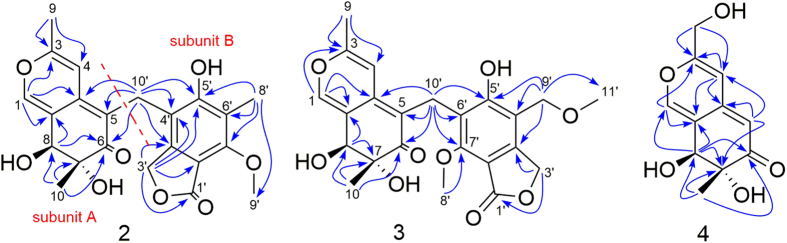
Key HMBC correlations of myxotritone (A–C)(2–4).

**Figure 5:**
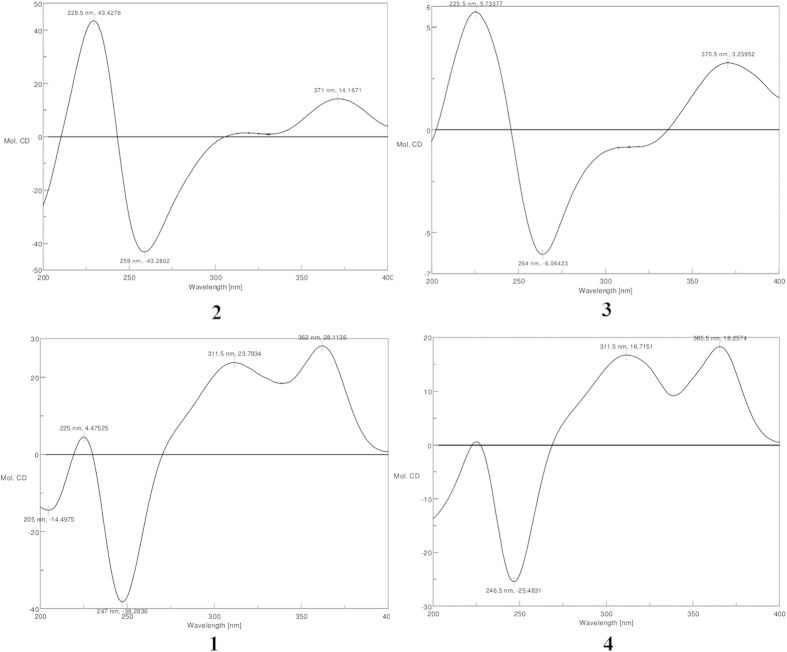
Experimental ECD of compounds (1–4).

Myxotritone A (**2**) was isolated as a yellow powder, [α]_D_^22^ = +45.6 (*c* = 0.125, MeOH). Its molecular formula was determined as C_22_H_22_O_8_ (12 degrees of unsaturation) by TOF-ESI-MS spectral data, which showed a pseudomolecular ion at *m/z* 437.1209 [M + Na]^+^ (Fig. S10). The UV spectrum of **2** displayed the maximum absorptions at 217 nm (log *ε* 4.21), 256 nm (log *ε* 3.86) and 364 nm (log *ε* 3.97) (Fig. S12), revealing the presence of an extended conjugated system as the characteristic of azaphilones. The ^1^H, ^13^C NMR and HMQC spectra revealed that **1** contained four methyls (one methoxyl group), two methylenes with one oxygenated, an oxymethine unit, an oxygenated quaternary carbon, 12 olefinic carbons, an ester carbonyl carbon, and a keto carbonyl group, which explained all carbon signals of **2**. Analysis of the ^1^H and ^13^C-NMR data of **2** revealed the same structural fragment (subunit A) as **1**, except the H-5 in **1** was replaced by other moiety in **2**, and this conclusion was supported by HMBC correlations (Fig. 4). The remaining connectivity was solved by detailed analysis of HMBC spectrum. The correlations from 10′-CH_2_- to C-3′a, C-4′ and C-5′, from 3′-CH_2_- to C-3′a, C-4′ and C-7′a, 8′-CH_3_ to C-5′, C-6′ and C-7′ together with correlations of the 9′-methoxyl with C-7′ established a hexa substituted phenyl ring. The key correlation from 3′-CH_2_- to the ester carbonyl and considering the chemical shift value of C-7′a (*δ*_C_ 107.8) led to construct an isobenzofuran-1(3*H*)-one fragment (subunit B). The correlations from 10′-CH_2_- to C-4a, C-5 and C-6 connected the subunit A with subunit B (Fig. 4). Considering the chemical shift values of C-7, C-8, and C-5′ and molecular formula, these three carbons must be anchored a free hydroxyl group, respectively. Thus the planar structure of **2** was determined. Compound **2** showed positive (371 nm, 311 nm and 229 nm) and negative (259 nm) cotton effects in the CD spectrum (Fig. 5). Based on the similar CD data and same biosynthetic pathway with **1**, the relative and absolute configurations of **2** were postulated to be 7*R*, 8*S*.

Myxotritone B (**3**) was obtained as yellow powder, [α]_D_^22^ = +6.0 (*c* = 0.067, MeOH). The molecular formula of **3** was deduced as C_23_H_24_O_9_ on the basis of its TOF-ESI-MS spectrum, in which a pseudomolecular ion was observed at *m/z* 467.1313 [M + Na]^+^ (Fig. S17). The ^1^H and ^13^C NMR spectra for **3** were similar with those of **2** except that one more methoxyl signal was observed, and the methyl anchored at the phenyl ring was disappeared in **3**, which implied that the methyl group on the phenyl ring in **3** was methoxylation. Yet, careful analysis of the ^1^H NMR of **2** and **3** revealed that the peak shape of 10′-CH_2_ in **2** and in **3** was completely different: singlet in **2**, whereas two doublets in **3**. This phenomenon implied that the substitutes around C-10′ in **3** were different from those in **2**, leading to the chemical environment change, which produced anisotropic characteristics of 10′-CH_2_ in **3**. Thus further HMBC spectrum was done to explain the phenomenon. The HMBC correlations clearly revealed that the connection of lactone ring was changed in **3** (Fig. 4), which finally established the planar structure of **3**. The relative and absolute configurations of **3** were postulated to be 7*R*, 8*S*, due to its similar CD data and same biosynthetic origin with **1**.

Myxotritone C (**4**) was obtained as yellow needle, [α]_D_^22^ = +403.3 (*c* = 0.15, MeOH). Its molecular formula was assigned as C_11_H_12_O_5_ (6 degrees of unsaturation) by TOF-ESI-MS spectral data, which showed a pseudomolecular ion at *m/z* 225.0760 [M + H]^+^, 247.0573 [M + Na]^+^ (Fig. S24). The ^1^H and ^13^C NMR spectra of **4** suggested the presence of similar subunit found in **1** except for 9-methyl signal (*δ*_C_ 17.9/*δ*_H_ 2.18) replaced by a methylol signal (*δ*_C_ 59.9/*δ*_H_ 4.29). This hypothesis was further confirmed by HMBC correlations (Fig. S23) (Fig. 4). The HMBC correlations from 9-CH_2_OH to C-3, C-4 and C-4a confirmed that the additional methylol group was connected with C-3. Similarly, the relative and absolute configurations of **4** were also deduced as 7S and 8S.

**Figure 6:**
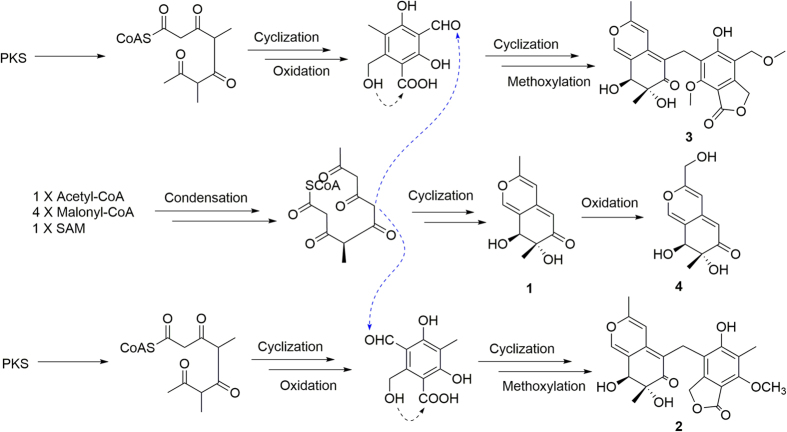
Possible pathway for the biosynthesis of (1–4).

From the structural features of compounds **1–4**, all these four compounds might come from same Non-Reduced Polyketide synthase (NR-PKS) origins. The putative biosynthetic pathway was suggested in the Fig. 6.

**Figure 7:**
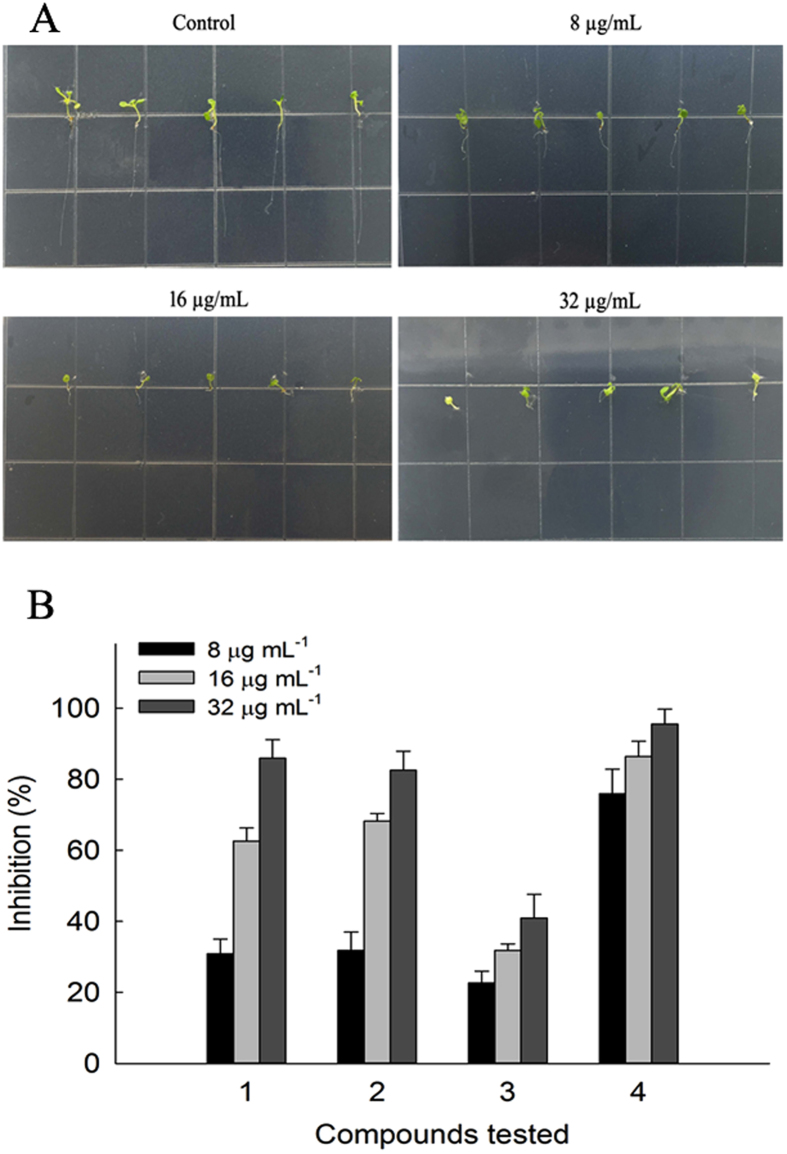
Effect of compounds (1–4) on seedling growth of *A. thaliana*. (**A**) The growth of *A.thaliana* on Petri dishes with different concentrations of compound (**4**) at 8, 16 and 32 *μ*g/mL, and DMSO was used as blank control with the same volume. (**B**) The inhibition on *A.thaliana* root growth of compounds (**1**–**4**) at different concentrations.

To study the potential effects of these metabolites, allelopathic potential of these compounds was tested with the root elongation of *A. thaliana* as model. The root growth of *A. thaliana* was inhibited after treatment with the compounds in a dose dependent manner (Fig. 7). The inhibition of compounds **1**–**4** at different concentrations was shown in Fig. 7. Compound **4** were found to retard the *Arabidopsis* seeds root significantly, with the inhibition rate of 75.9% at 8 *μ*g/mL, whereas compounds **1** and **2** showed moderate inhibition activities. The results implied that this fungus might contribute to the defense of its host lichen.

## Methods

### General Experimental Procedures

Optical rotations were measured on a Perkin-Elmer 241 Polarimeter (Perkin-Elmer, Bruker, Billerica, MA, USA) and UV data were obtained on a Shimadzu Biospec-1601 spectrophotometer. CD spectra were obtained on a Chirascan spectropolarimeter. ^1^H and ^13^C NMR data were acquired using Bruker 600 and Varian Inova 600 spectrometers using solvent signals (MeOH-*d*_4_; *δ*_H_ 4.87, 3.31/*δ*_C_ 49.15) as references. HRESIMS data were acquired using a LTQ Orbitrap XL Mass Spectrometer (Thermo, Waltham, MA, USA).

### Fungus and culturing condition

The endolichenic fungus *Myxotrichum* sp. was isolated from the lichen *Cetraria islandica* (L.) Ach. collected from Laojun Mountain, Yunnan Province, People’s Republic of China. The isolate was identified on the basis of the internal transcribed spacer region sequences of the rDNA (Genbank Accession No. HQ324780) (Fig. S1) and the fungus assigned the accession no.20081189 was deposited at lichen laboratory’s culture collection in College of Life Sciences, Shandong Normal University, Jinan. The fungal strain was cultured on slants of potato dextrose agar (PDA) at 25 °C for 15 days. Then, the proper fungus strain was inoculated in five Erlenmeyer flasks (500 mL) each containing 200 mL PDB (20% potato and 2% glucose) media. Flask cultures were incubated at 25 °C on a rotary shaker at 110 rpm for seven days as spore seeds. These spore seeds were used to inoculate in Fernbach flasks (500 mL), each containing 60 g of rice, and incubated at 25 °C for 40 days.

### Extraction and isolation

The fermented material was extracted with ethyl acetate (6 L for four times). The solution was concentrated to dryness under vacuum to afford a crude extract (22.0 g), which was fractionated by silica gel column chromatography (10 × 100 cm) using CH_2_Cl_2_–MeOH gradient elution. The fraction (611 mg) eluted with CH_2_Cl_2_–MeOH 50:1 was separated by Sephadex LH-20 (Pharmacia, Uppsala, Sweden) column chromatography eluting with MeOH to afford 4 subfractions. The resulting subfraction 1 was further purified by semipreparative RP HPLC (Lumtech, Berlin, Germany; YMC-Pack ODS-A column; 10 *μ*m; 250 × 10 mm; 2 mL · min^−1^, 46% MeOH in H_2_O) to afford **1** (30 mg, t_R_ = 14.3 min), subfraction 4 was purified by RP-HPLC (Lumtech, Berlin, Germany; YMC-Pack ODS-A column; 10 *μ*m; 250 × 10 mm; 2 mL · min^−1^, 70% MeOH in H_2_O for 20 min) to afford myxotritone A (**2**, 4 mg, t_R_ = 10 min) and myxotritone B (**3**, 1 mg, t_R_ = 13 min). Fraction (863 mg) eluted with CH_2_Cl_2_–MeOH 10:1 were fractionated again by Sephadex LH-20 column chromatography using MeOH as eluent. Purification of the subfraction by RP-HPLC (Lumtech; YMC-Pack ODS-A column; 10 *μ*m; 250 × 10 mm; 2 mL · min^−1^,12% MeOH in H_2_O) afforded myxotritone C (**4**, 6 mg, t_R_ = 14 min).

*7R, 8S -7, 8-dihydro-7, 8-dihydroxy-3, 7-dimethyl-2- benzopyran-6-one* (**1**): Brown powder, [α]_D_^22^ = +393.0° (*c* 0.15, MeOH); UV(MeOH) *λ*_max_ (log *ε*) 349 (4.45) nm; CD (MeOH) 205 (Δ*ε*-0.21), 225 (Δ*ε* + 0.06), 247 (Δ*ε*−0.55), 311 (Δ*ε* + 0.34), 362 (Δ*ε* + 0.40) nm; IR (KBr) *V*max: 3430, 2981, 2937, 1717, 1645, 1602, 1546, 1457, 1394, 1369, 1326, 1087, 1298, 990 967, 893 cm^−1^; ^1^H NMR and ^13^C NMR, see Table 1; positive HRESIMS *m/z* 209.0813 [M + H]^+^ (calcd for C_11_H_13_O_4_, 209.0814).

*Myxotritone A (**2**):* Yellow needle, [α]_D_^22^ = +45.6 (*c* 0.125, MeOH), UV (MeOH) *λ*_max_ (log *ε*) 217 (4.21) nm; 256 (3.86) nm; 364 (3.97) nm; CD (MeOH) 230 (Δ*ε* + 1.47), 259 (Δ*ε* −1.47), 371 (Δ*ε* + 0.48) nm; IR (KBr) *V*max: 3436, 2951, 2843, 1732, 1674, 1661, 1599, 1485, 1329, 1279, 1187, 1146, 1098, 1020, 962, 909, 879, 847, 825 cm^−1^; ^1^H NMR and ^13^C NMR, see Table 1; positive HRESIMS *m/z* 415.1394 [M + H]^+^ (calcd for C_22_H_23_O_8_, 415.1393), 437.1209 [M + Na] ^+^ (calcd for C_22_H_23_O_8_Na, 437.1212).

*Myxotritone B (**3**):* Yellow powder, [α]_D_^22^ = +6.0° (*c* 0.067, MeOH); UV (MeOH) *λ*_max_(log *ε*) 221 (3.91), 256 (3.56) and 370 (3.61) nm; CD (MeOH) 226 (Δ*ε* + 0.77), 264 (Δ*ε* -0.82), 371 (Δ*ε* + 0.44) nm; IR (KBr) *V*max: 3436, 2954, 2844, 1636, 1517, 1457, 1448, 1387, 1350, 1339, 1277, 1187, 1101, 1064, 1032, 1017, 966 cm^−1^; ^1^H NMR and ^13^C NMR, see Table 1; positive HRESIMS *m/z* 467.1313 [M + H]^+^ (calcd for C_23_H_25_O_9_Na, 467.1318).

*Myxotritone C (**4**):* Brown powder, [α]_D_^22^ = +403.3° (*c* 0.15, MeOH); UV (MeOH) *λ*_max_(log *ε*) 233 (3.86), 243 (3.83) and 348 (4.46) nm; CD (MeOH) 247 (Δ*ε* -1.94), 312 (Δ*ε* + 1.28), 366 (Δ*ε* + 1.39) nm; IR (KBr) *V*max: 3430, 2913, 2864, 1674, 1653, 1623, 1616, 1545, 1512, 1444, 1398, 1373, 1326, 1270, 1235, 1160, 1123, 1061, 1015, 970, 930, 883, 830 cm^−1^; ^1^H NMR and ^13^C NMR, see Table 1; positive HRESIMS *m/z* 225.0760 [M + H]^+^ (calcd for C_11_H_13_O_5_, 225.0762).

### Seedling growth test

*Arabidopsis thaliana* seeds were surface sterilized by 5% sodium hypochlorite for 5 min, followed by washing with sterile distilled water for five times. Compounds were dissolved with DMSO to final concentration of 40 mg/mL. Then 20 *μ*L of them were added to 25 mL 1/2 MS medium supplemented with 0.8% (w/v) agar to get plates with different concentrations of compounds (8, 16, 32 *μ*g/mL). To eliminate the effect of DMSO on the growth of *A. thaliana*, plates with 20 *μ*L DMSO were used as blank control. Fifteen seeds were distributed on each Petri dishes described before. Each concentration was conducted in triplicate. The Petri dishes were placed in a growth chamber at 23 ± 1 °C under light for 8 h and darkness for 6 h. The lengths of roots were measured after 9 days. The percentage of growth inhibition of root lengths was calculated as the following equation:


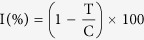


where T stands for the average length of treatment (cm) and C stands for the average length of control (cm)^14^,^15^.

## Additional Information

**How to cite this article**: Yuan, C. *et al*. Allelopathic Polyketides from an Endolichenic Fungus *Myxotrichum* SP. by Using OSMAC Strategy. *Sci. Rep*. **6**, 19350; doi: 10.1038/srep19350 (2016).

## Supplementary Material

Supplementary Information

## Figures and Tables

**Figure 1:**
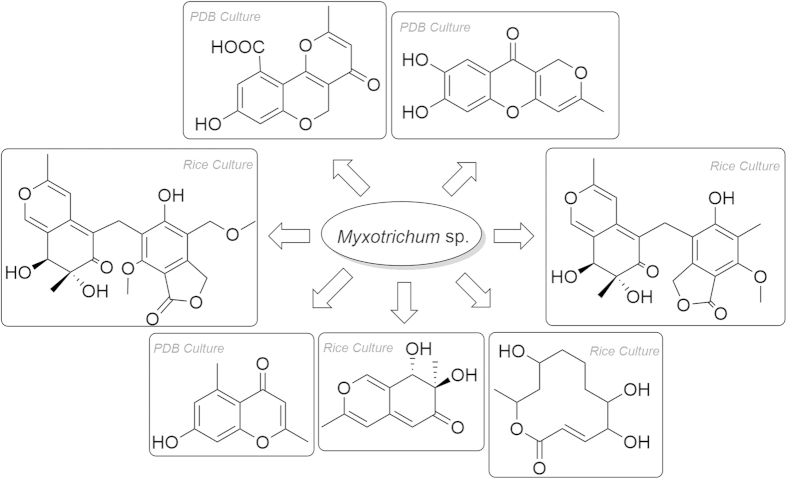
Diverse skeleton polyketides isolated from the endolichenic fungus *Myxotrichum* sp.

**Table 1:** ^1^H (600MHz) and ^13^C NMR (150 MHz) spectroscopic data (MeOH-*d*_4_) of myxotritone A–C (2–4).

No.	2		3		4	
	*δ*_C_ [ppm]	*δ*_H_ [ppm], M(*J* in Hz)	*δ*_C_ [ppm]	*δ*_H_ [ppm], M(*J* in Hz)	*δ*_C_ [ppm]	*δ*_H_ [ppm], M(*J* in Hz)
1	145.3	7.48, d (1.8)	145.9	7.57, d (1.8)	145.3	7.47, t (1.2)
2						
3	161.1		161.6		162.3	
4	104.2	6.69, s	105.3	7.07, s	104.9	6.39, s
5	111.6		111.9		104.7	5.37, d (1.2)
6	199.4		201.1		200.1	
7	76.0		76.0		76.5	
8	71.5	4.44, d (2.4)	71.4	4.47, d (1.8)	72.1	4.54, d (2.4)
9	18.4	2.28, s	18.6	2.34, s	59.9	4.29, s
10	17.5	1.11, s	17.5	1.15, s	17.7	1.16, s
4a	144.3		146.2		146.7	
8a	121.1		121.5		121.4	
1′	170.5		170.2			
3′	69.1	5.28, d (15.6) 5.15, d (15.6)	69.0	5.32, d (15.6) 5.28, d (15.6)		
4′	116.8		115.7			
5′	161.2		160.7			
6′	119.4		120.5			
7′	156.3		157.5			
8′	7.9	2.16, s	61.7	4.05, s		
9′	61.0	3.90, s	66.8	4.60, s		
10′	21.1	3.59, s	19.0	3.79, d (14.4) 3.53, d (14.4)		
11′			57.3	3.40, s		
3′a	147.2		148.2			
7′a	107.8		107.9			
