# Supplementary material for: Allelopathic Polyketides from an Endolichenic Fungus Myxotrichum SP. by Using OSMAC Strategy
Source: Sci Rep. 2016 Feb 3;6:19350. doi: 10.1038/srep19350 (PMC4738244; doi:10.1038/srep19350)

SUPPLEMENTARY INFORMATION

DOI:

Title: **Allelopathic Polyketides from an Endolichenic Fungus *Myxotrichum* SP. by Using OSMAC Strategy**

Author(s): Chao Yuan [1,‡], Yu-Hua Guo [2,‡], Hai-Ying Wang [3], Xiao-Jun Ma [2], Tao Jiang [2], Jun-Ling Zhao [1], Zhong-Mei Zou [2,]* & Gang Ding [2,] *

1. Institute of Medicinal Plant Development Yunnan Branch, Chinese Academy of Medical Sciences and Peking Union Medical College, Jinghong, P.R. China;

2. Institute of Medicinal Plant Development, Chinese Academy of Medical Sciences and Peking Union Medical College, Beijing, P.R. China;

3. College of Life Sciences, Shandong Normal University, No. 88 East Wenhua Road, Jinan, P.R. China;

[‡]These authors contributed equally to the work. Correspondence and requests for materials should be addressed to Zhongmei Zou (email: zmzou@implad.ac.cn) or Gang Ding (email: gding@implad.ac.cn)

Table of contents

SI-3	Fig. S1	The systematic position of endolichenic fungus 081189
SI-4	Fig. S2	^{1}H NMR spectrum (600 MHz) of **1** in Methanol-d_4
SI-4	Fig. S3	^{13}C NMR spectrum (150 MHz) of **1** in Methanol-d_4
SI-5	Fig. S4	CD and Mo-CD spectrum of 1
SI-6	Fig. S5	X-ray of 1
SI-7	Fig. S6	^{1}H NMR spectrum (600 MHz) of **2** in Methanol-d_4
SI-7	Fig. S7	^{13}C NMR spectrum (150 MHz) of **2** in Methanol-d_4
SI-8	Fig. S8	HSQC spectrum of **2** in Methanol- d_4
SI-8	Fig. S9	HMBC spectrum of **2** in Methanol-d_4
SI-9	Fig. S10	HRESIMS spectrum of **2**
SI-9	Fig. S11	IR (KBr disc) spectrum of 2
SI-10	Fig. S12	CD and UV spectra of **2**
SI-11	Fig. S13	^{1}H NMR spectrum (600 MHz) of **3** in Methanol-d_4
SI-11	Fig. S14	^{13}C NMR spectrum (150 MHz) of **3** in Methanol-d_4
SI-12	Fig. S15	HSQC spectrum of **3** in Methanol- d_4
SI-12	Fig. S16	HMBC spectrum of **3** in Methanol-d_4
SI-13	Fig. S17	HRESIMS spectrum of **3**
SI-13	Fig. S18	IR (KBr disc) spectrum of 3
SI-14	Fig. S19	CD and UV spectra of **3**
SI-15	Fig. S20	^{1}H NMR spectrum (600 MHz) of **4** in Methanol-d_4
SI-15	Fig. S21	^{13}C NMR spectrum (150 MHz) of **4** in Methanol-d_4
SI-16	Fig. S22	HSQC spectrum of **4** in Methanol- d_4
SI-16	Fig. S23	HMBC spectrum of **4** in Methanol-d_4
SI-17	Fig. S24	HRESIMS spectrum of **4**
SI-17	Fig. S25	IR (KBr disc) spectrum of 4
SI-18	Fig. S26	CD and UV spectra of **4**

Fig. S1 The systematic position of the endolichenic fungus 081189.

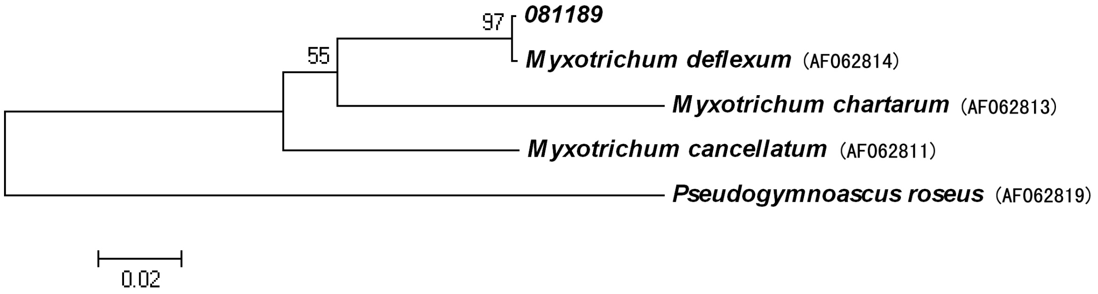

The systematic position of the endolichenic fungus 081189.

The phylogenetic tree based on the nuclear ITS rDNA sequences was inferred using the Neighbor-Joining method [1]. The percentage of replicate trees in which the associated taxa clustered together in the bootstrap test (1000 replicates) are shown next to the branches [2]. The evolutionary distances were computed using the Maximum Composite Likelihood method [3] and are in the units of the number of base substitutions per site. All positions containing alignment gaps and missing data were eliminated only in pairwise sequence comparisons (Pairwise deletion option). There were a total of 546 positions in the final dataset. Phylogenetic analyses were conducted in MEGA4 [4]. The numbers in brackets in the phylogenetic tree are the GenBank accession numbers of corresponding samples.

1. Saitou N & Nei M (**1987**) The neighbor-joining method: A new method for reconstructing phylogenetic trees. *Molecular Biology and Evolution* **4**:406-425.
2. Felsenstein J (**1985**) Confidence limits on phylogenies: An approach using the bootstrap. *Evolution* **39**:783-791.
3. Tamura K, Nei M & Kumar S (**2004**) Prospects for inferring very large phylogenies by using the neighbor-joining method. *Proceedings of the National Academy of Sciences* (*USA*) **101**:11030-11035.
4. Tamura K, Dudley J, Nei M & Kumar S (**2007**) MEGA4: Molecular Evolutionary Genetics Analysis (MEGA) software version 4.0. *Molecular Biology and Evolution* **24**:1596-1599.

Fig. S2 ^{1}H NMR spectrum (600 MHz) of **1** in Methanol-d$_4$

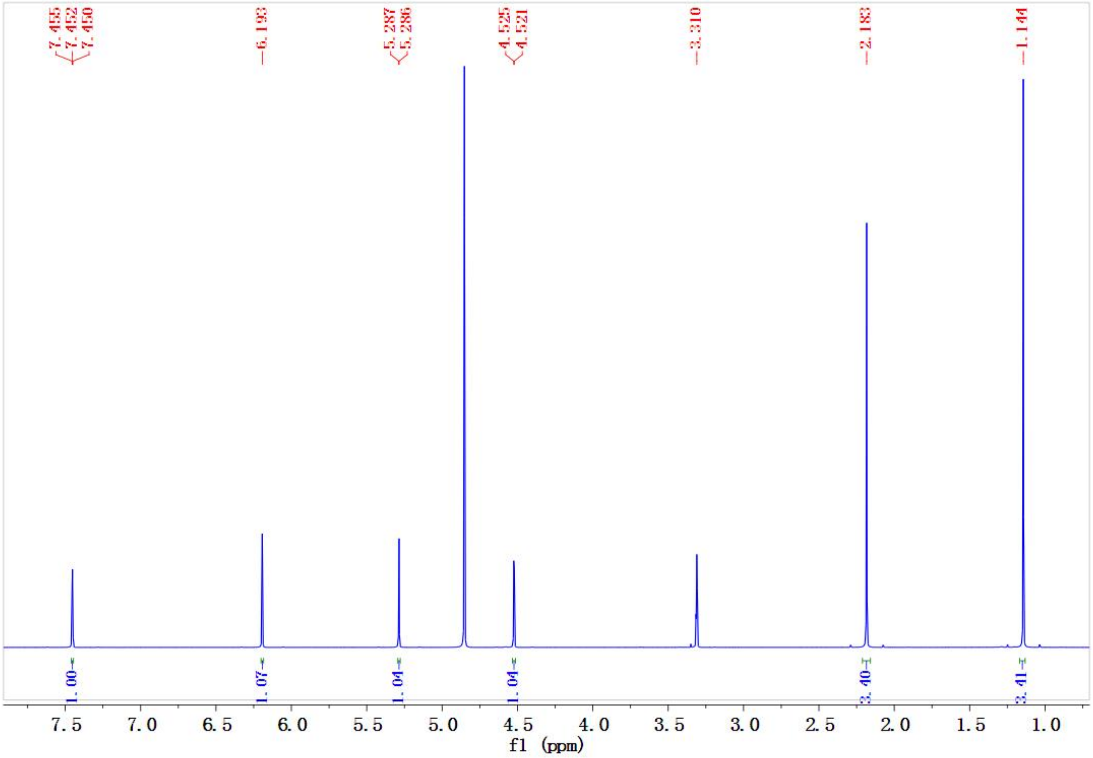

Fig. S3 ^{13}C NMR spectrum (150 MHz) of **1** in Methanol-d$_4$

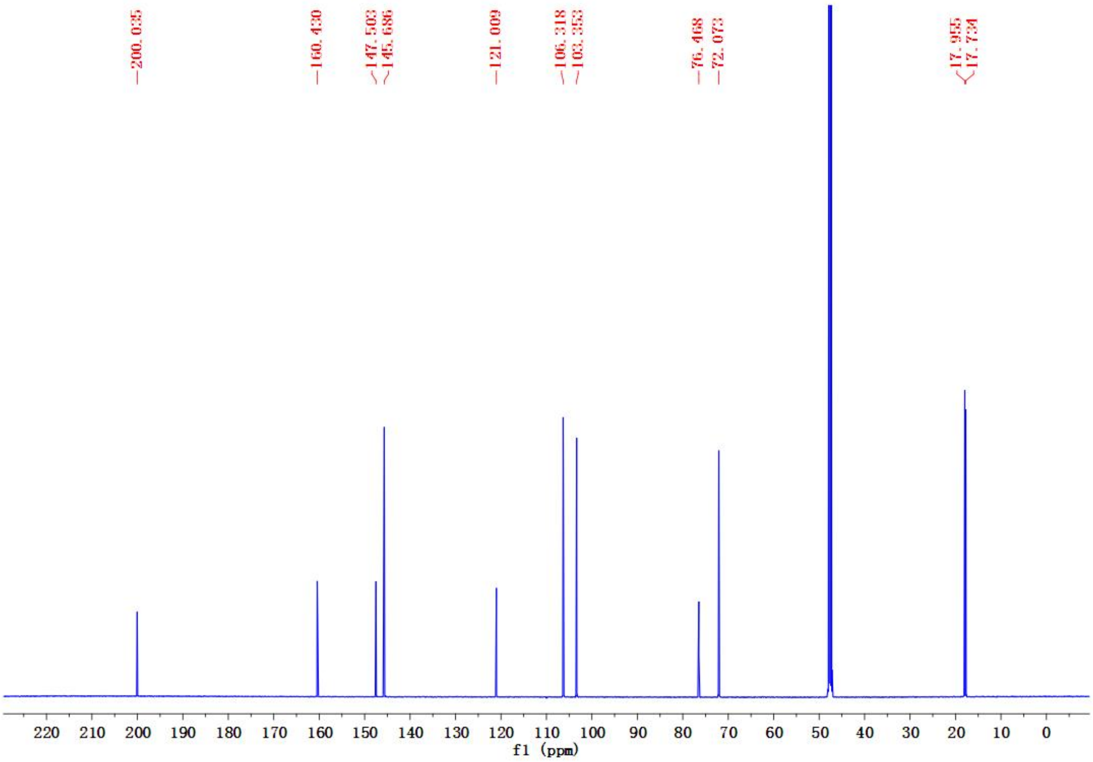

Fig. S4 CD and Induced-CD spectrum of 1

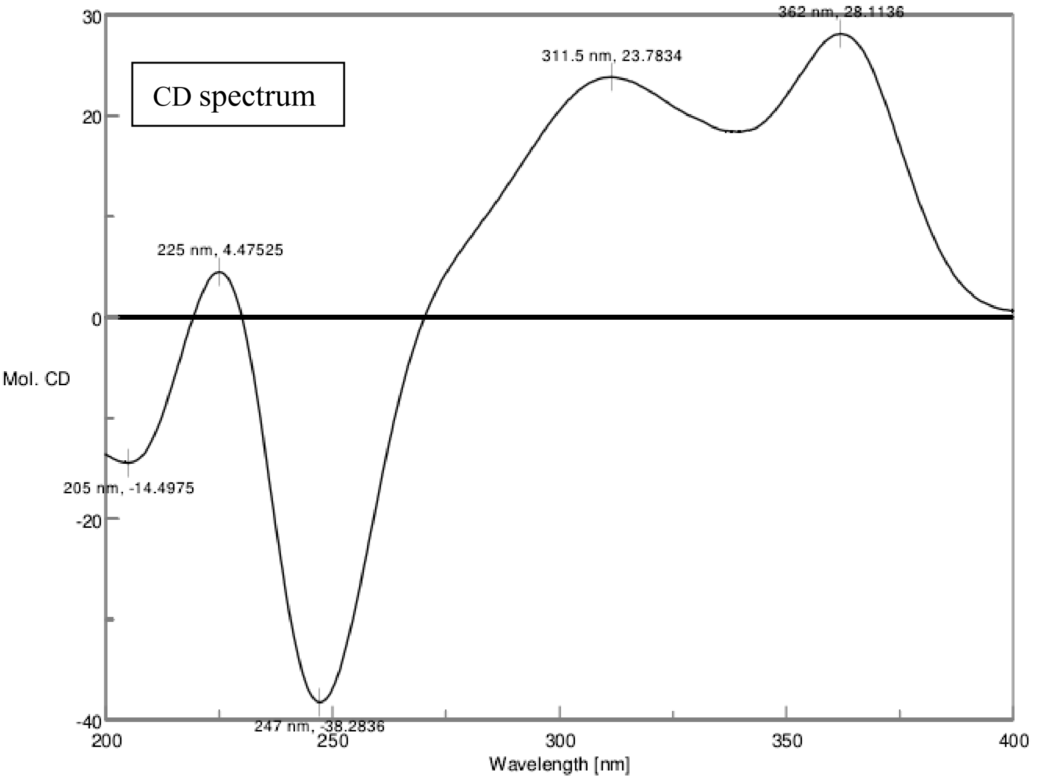

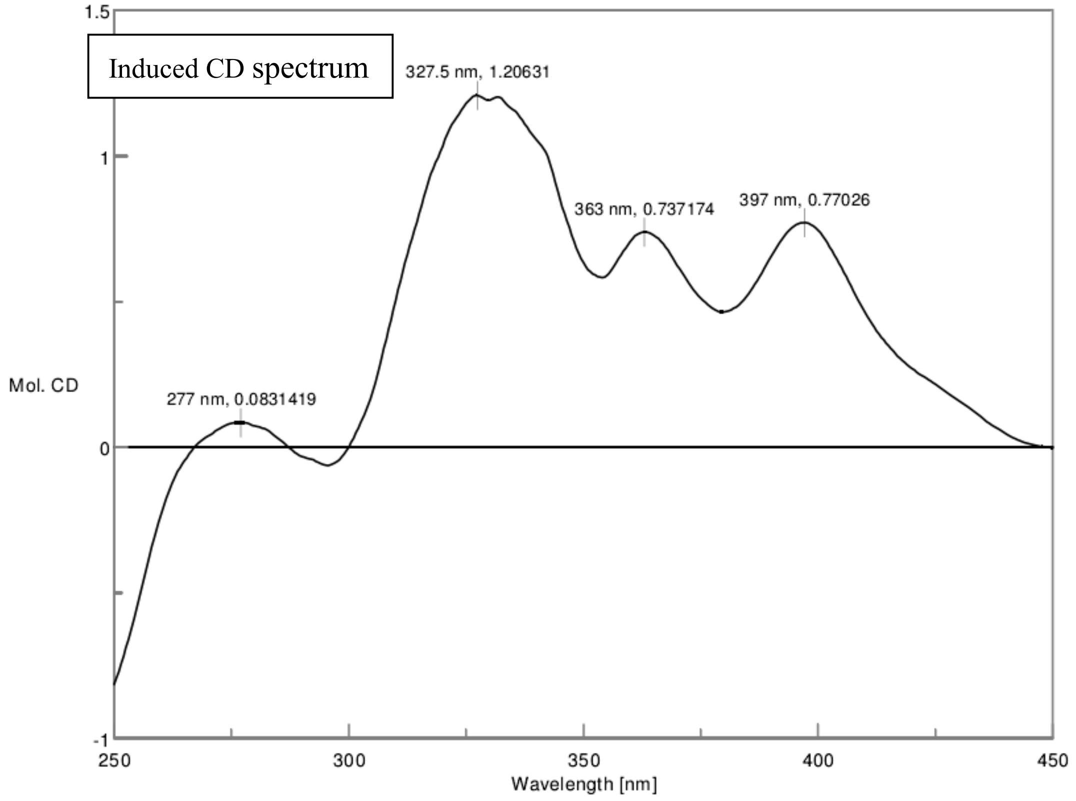

Fig. S5 X-ray of 1

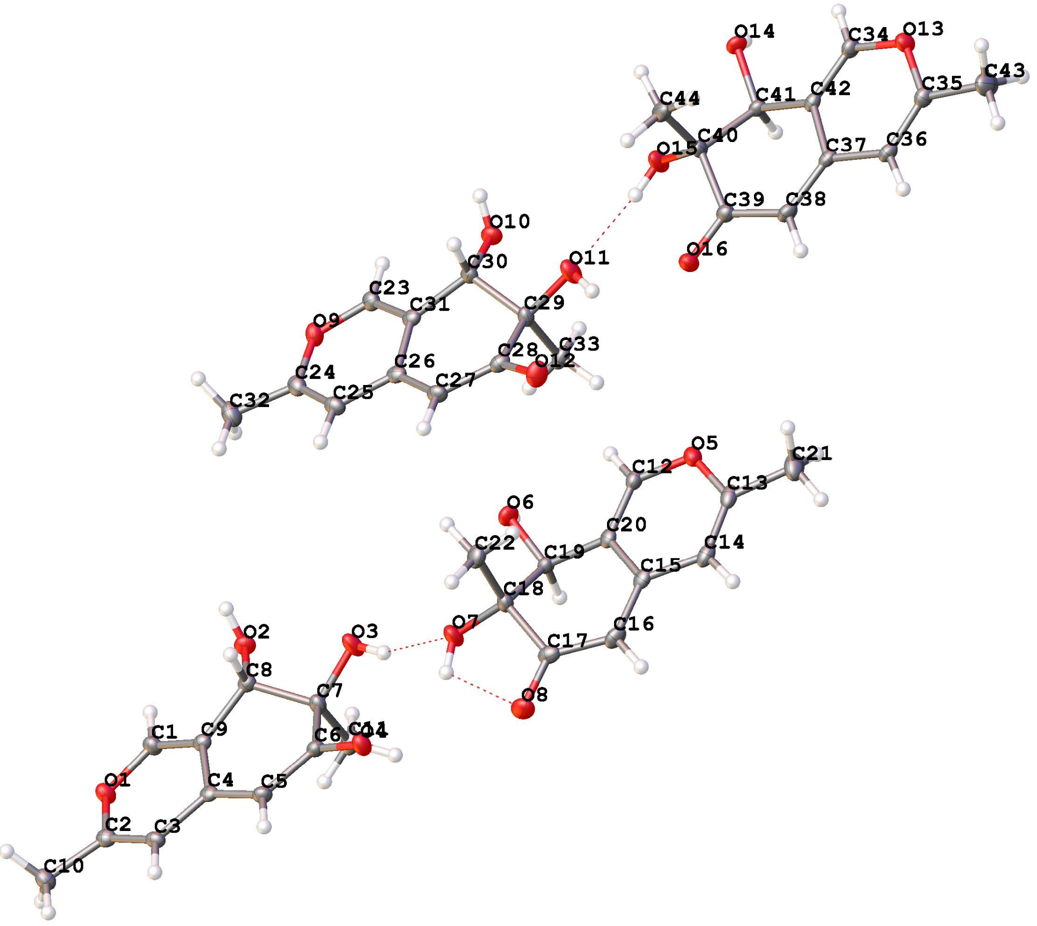

Fig. S6 ^{1}H NMR spectrum (600 MHz) of **2** in Methanol-d_4

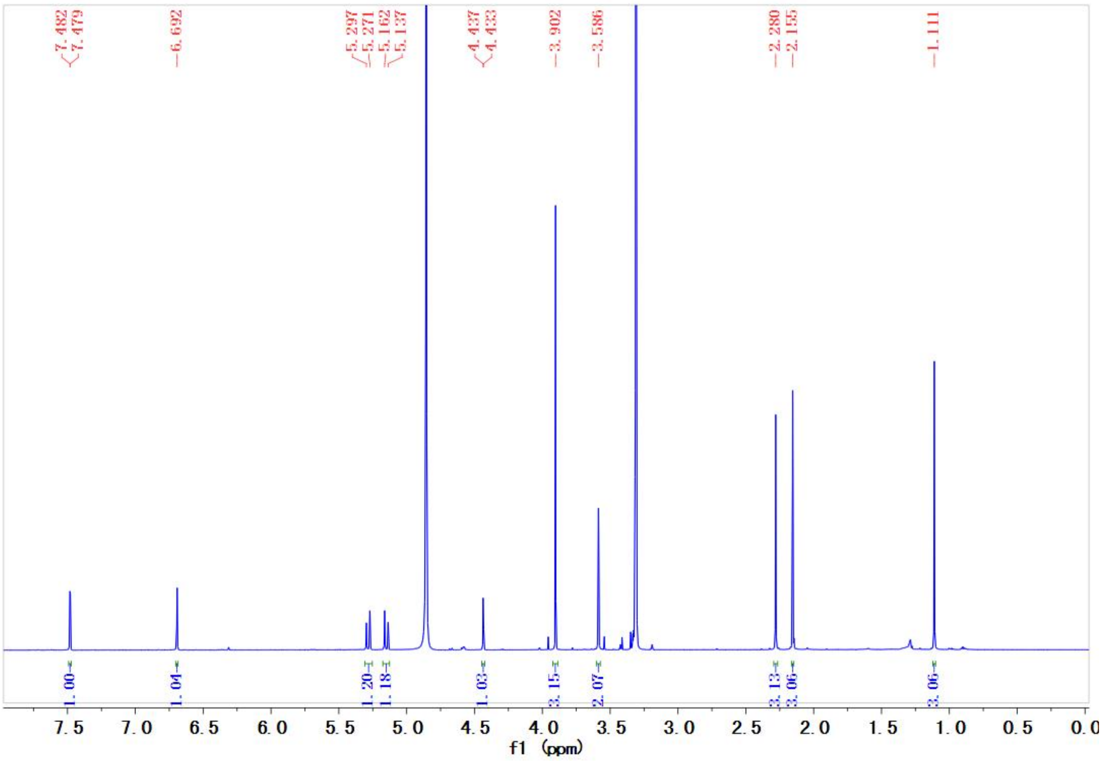

Fig. S7 ^{13}C NMR spectrum (150 MHz) of **2** in Methanol-d_4

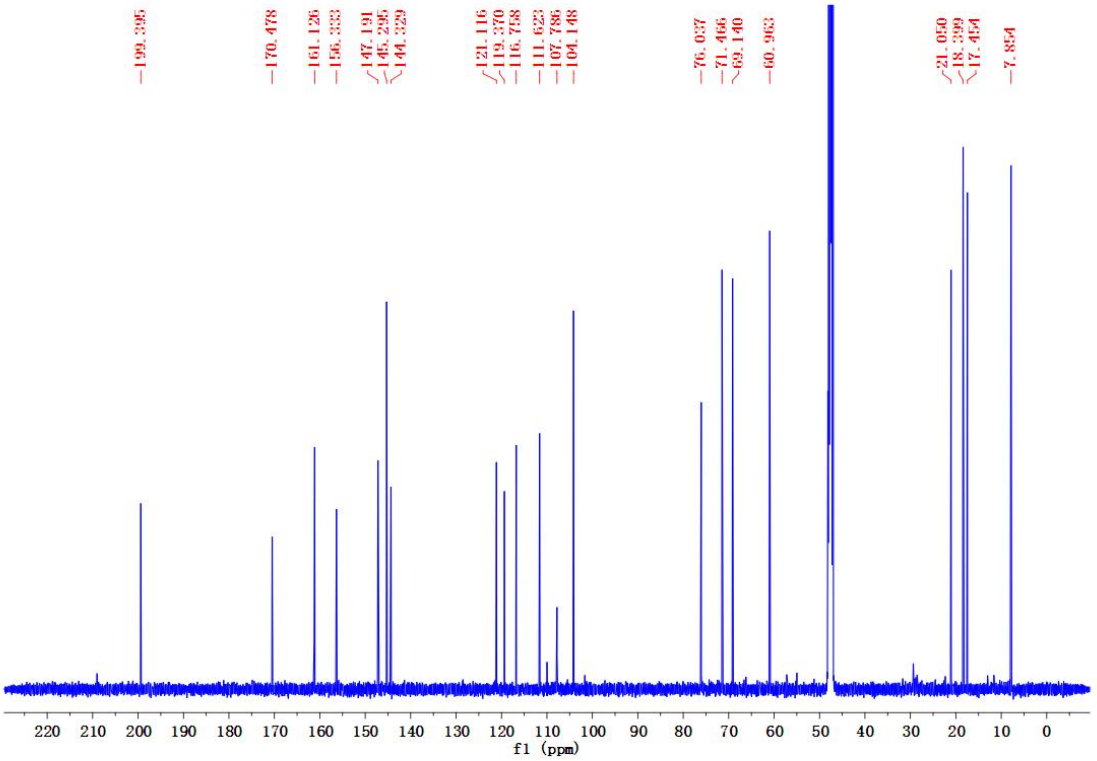

Fig. S8 HSQC spectrum of **2** in Methanol- d_4

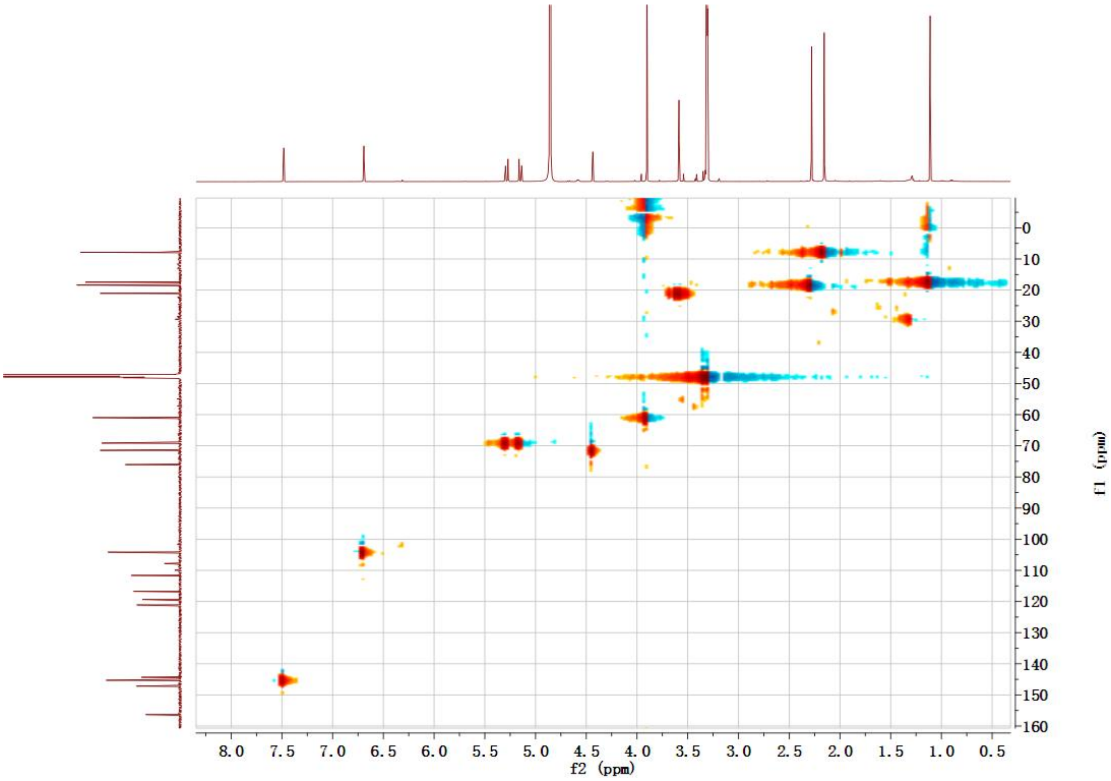

Fig. S9 HMBC spectrum of **2** in Methanol- d_4

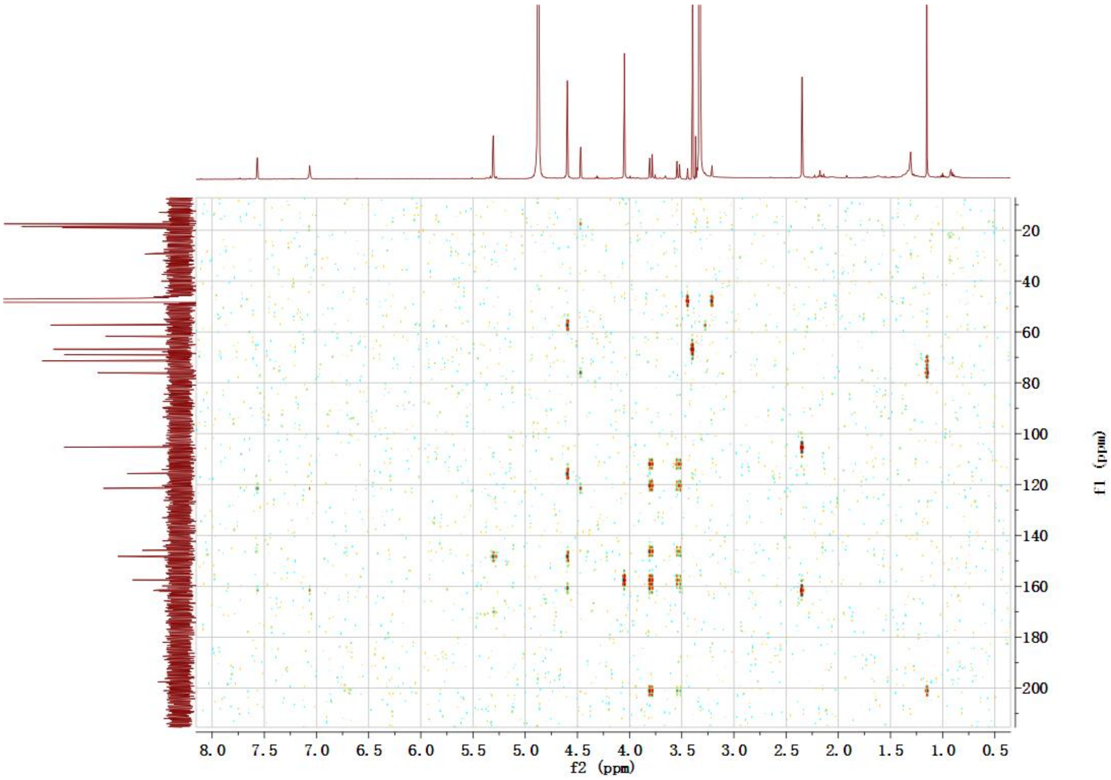

Fig. S10 HRESIMS spectrum of **2**

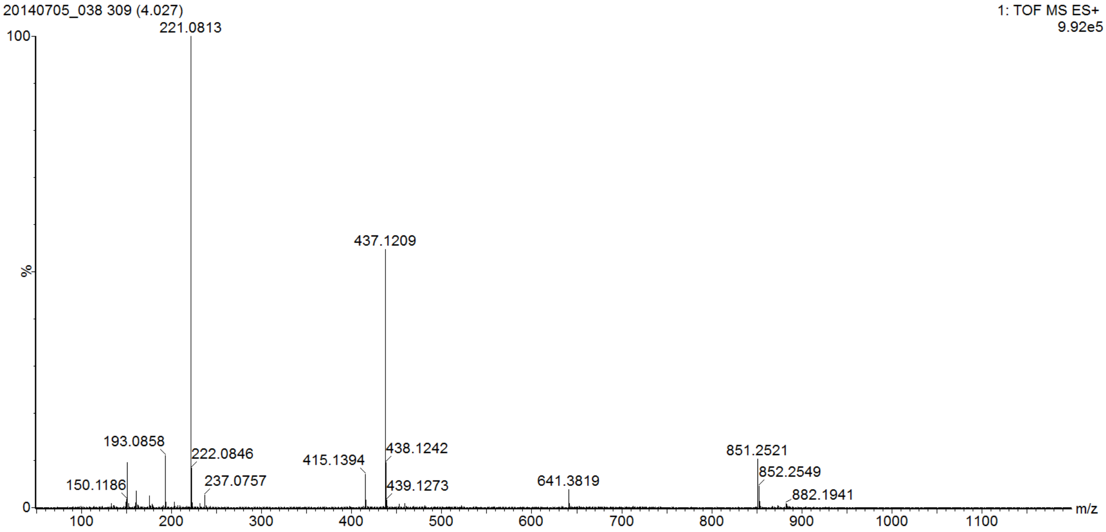

Fig. S11 IR (KBr disc) spectrum of 2

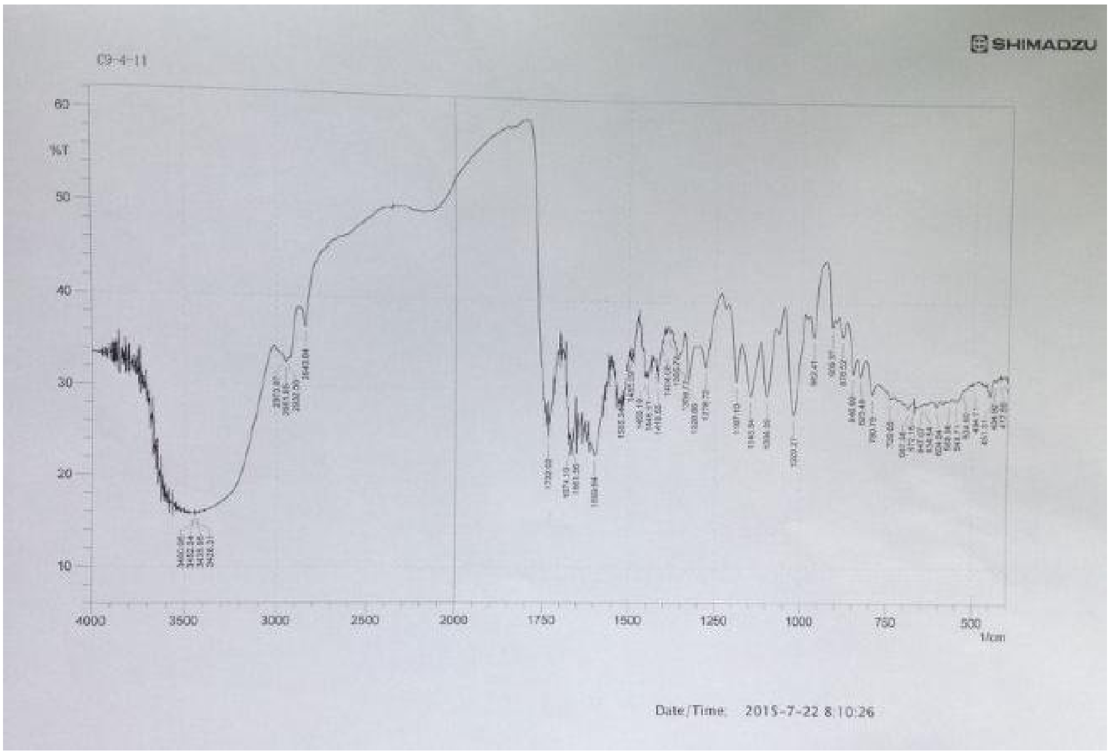

Fig. S12 CD and UV spectra of **2**

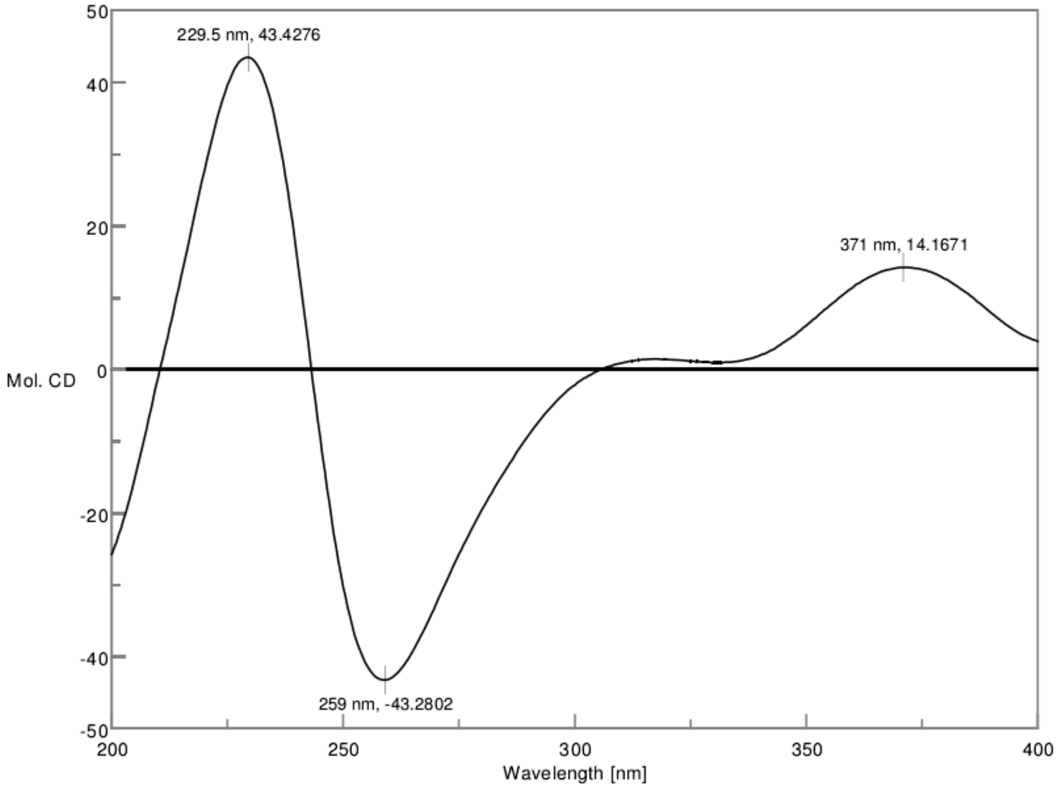

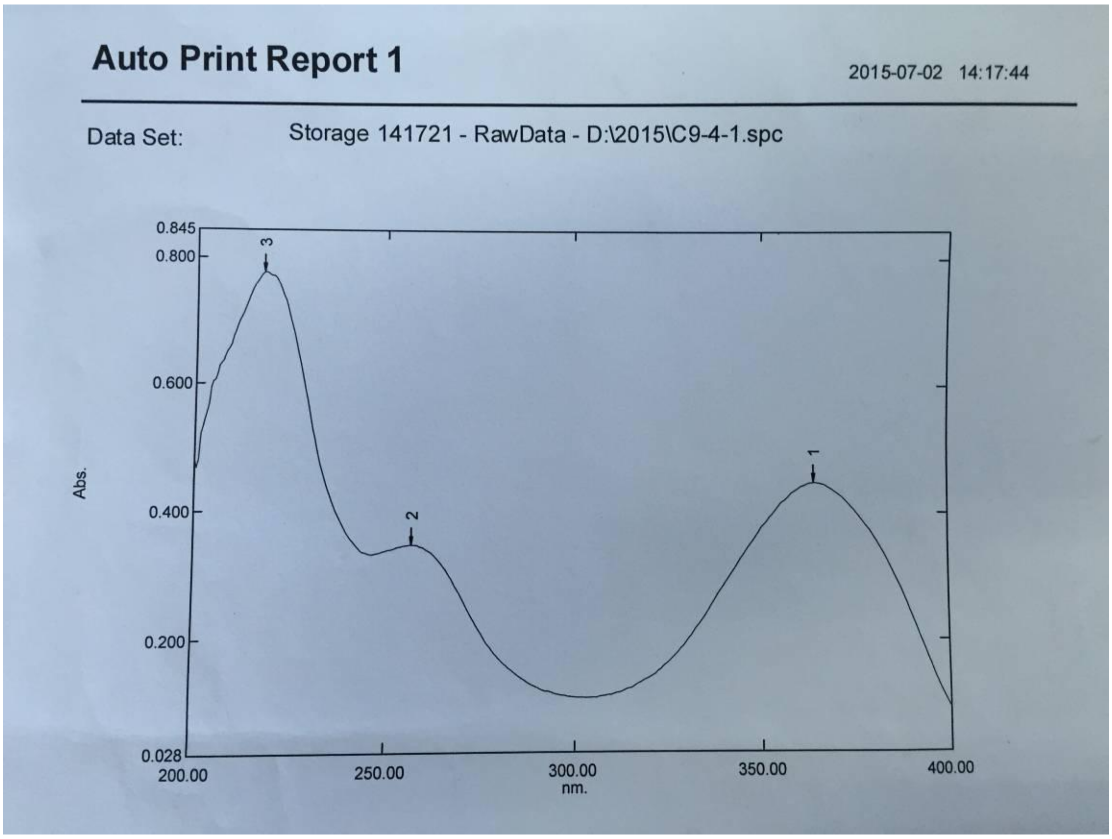

Fig. S13 ^{1}H NMR spectrum (600 MHz) of **3** in Methanol-d_4

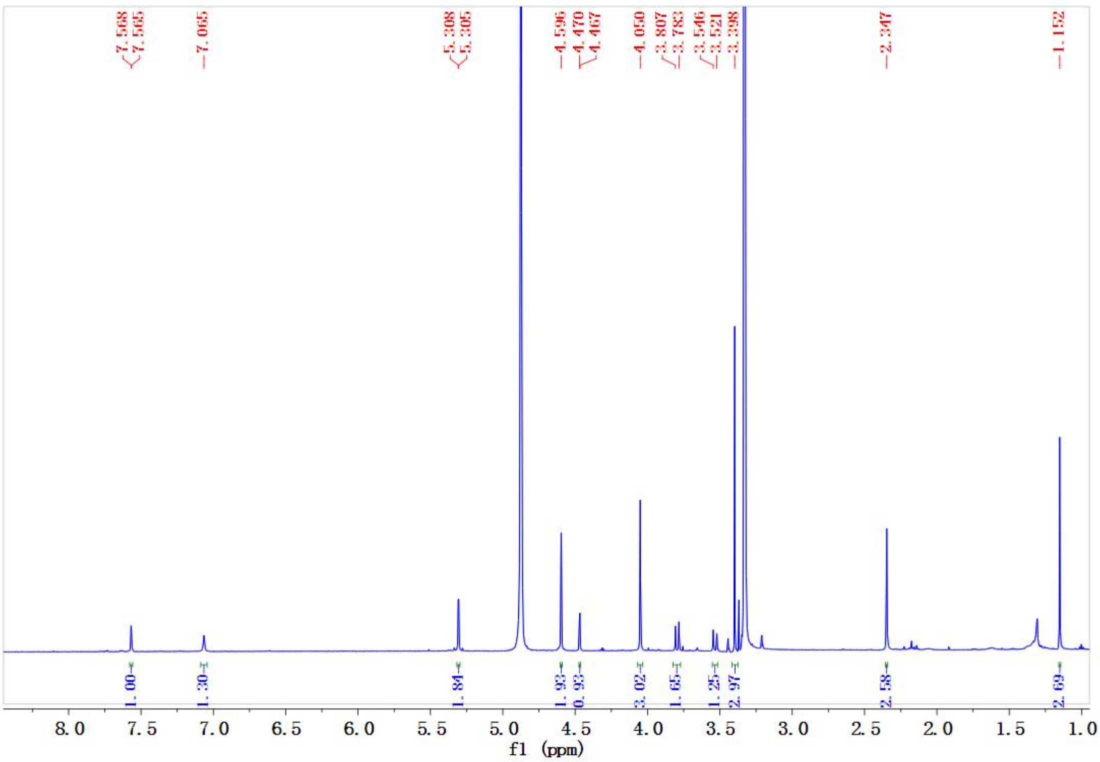

Fig. S14 ^{13}C NMR spectrum (150 MHz) of **3** in Methanol-d_4

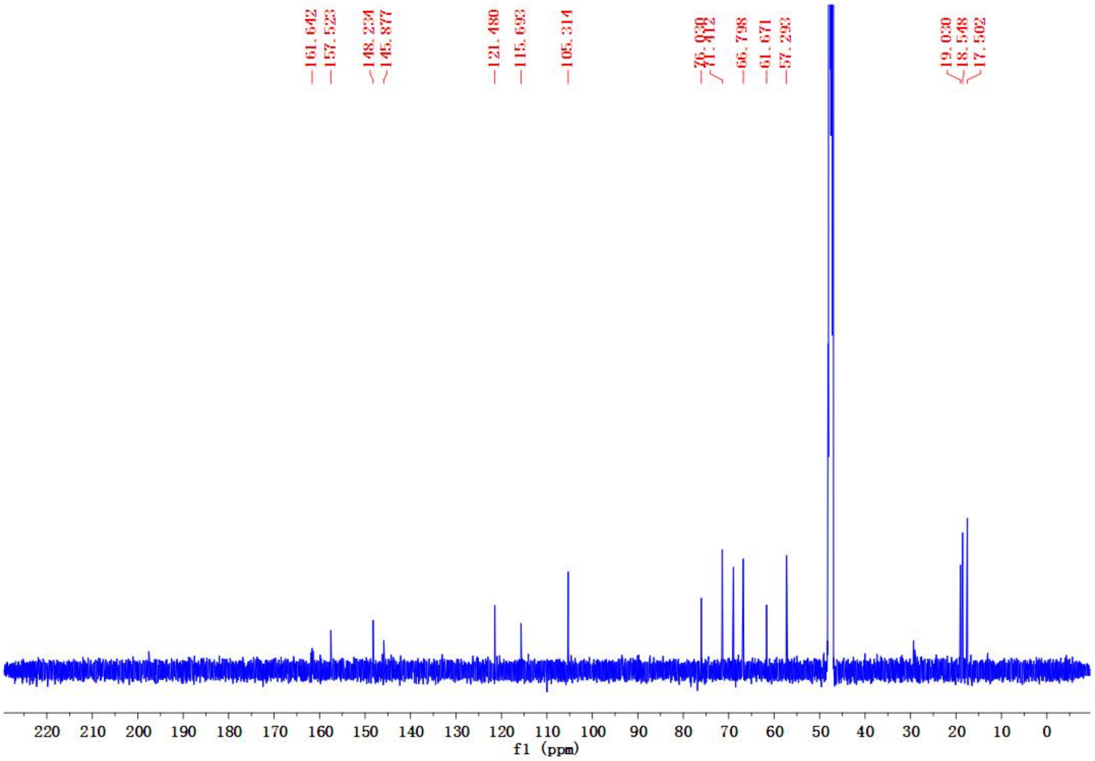

Fig. S15 HSQC spectrum of **3** in Methanol- d_4

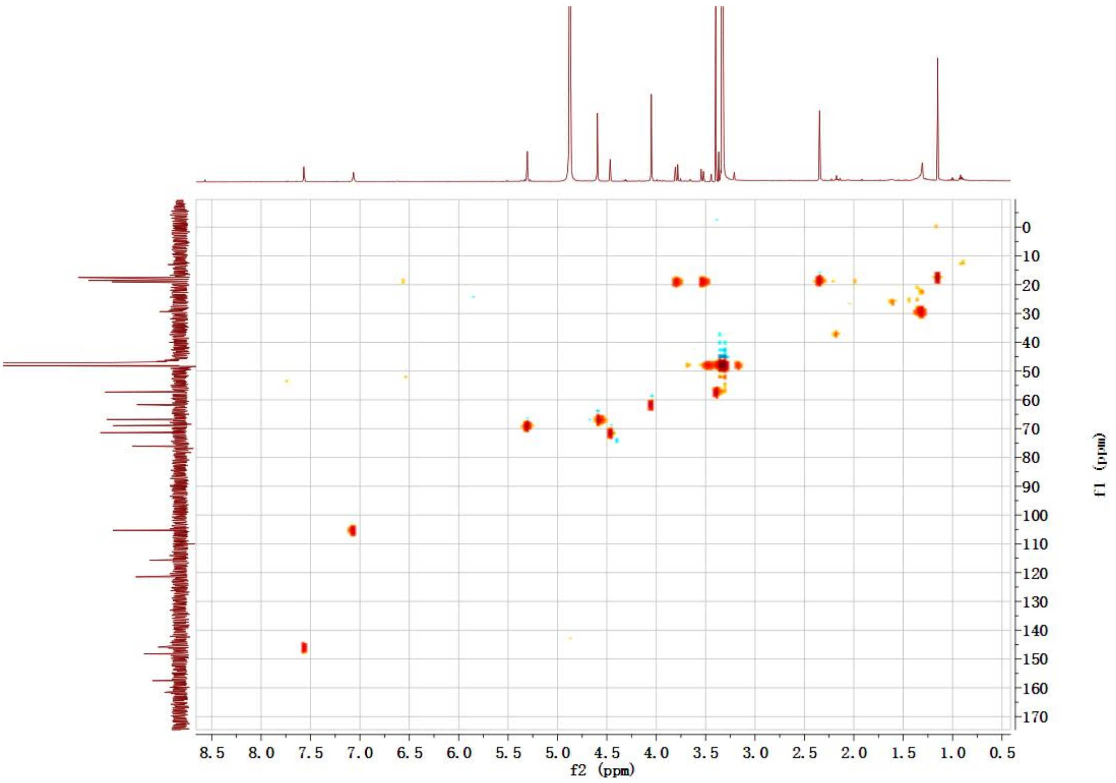

Fig. S16 HMBC spectrum of **3** in Methanol-d_4

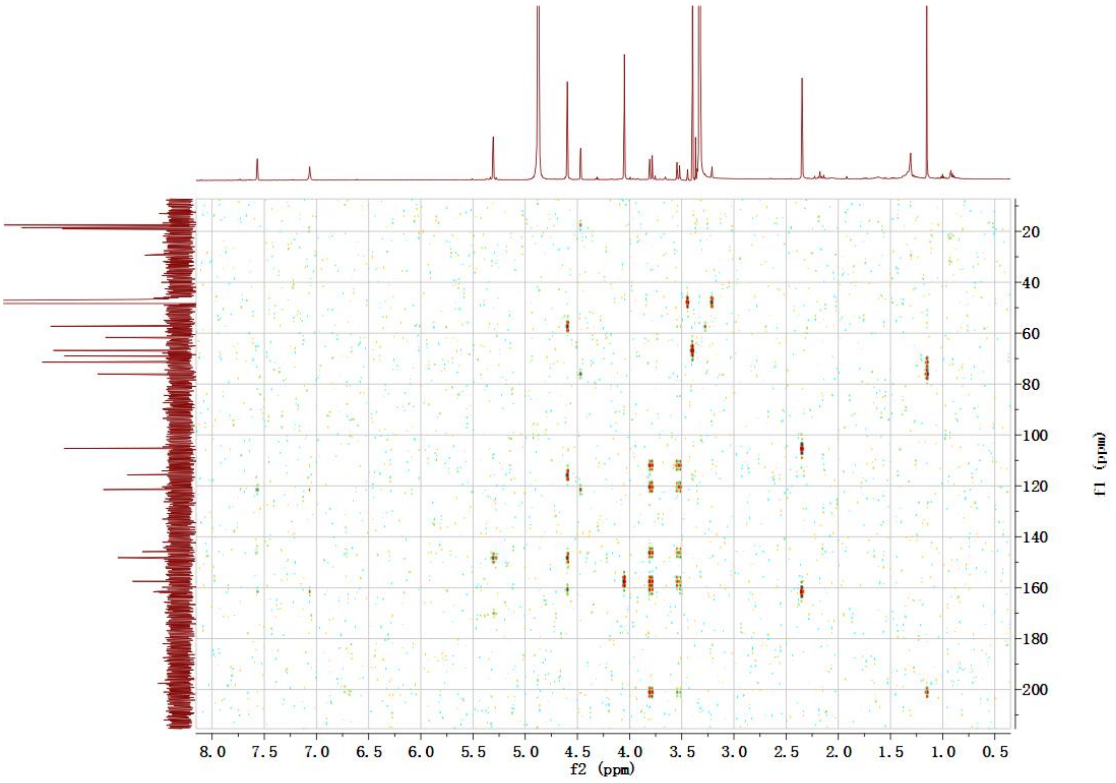

Fig. S17 HRESIMS spectrum of **3**

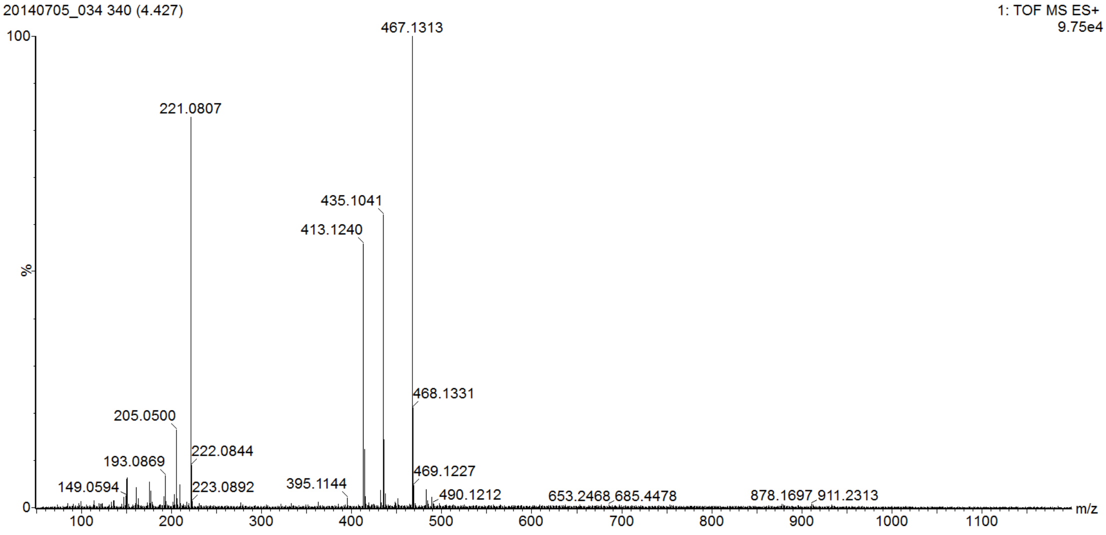

Fig. S18 IR (KBr disc) spectrum of 3

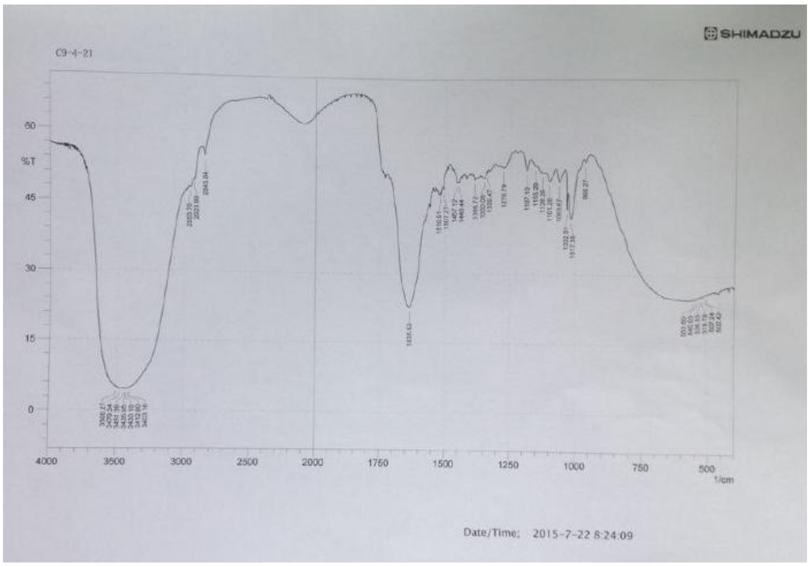

Fig. S19 CD and UV spectra of **3**

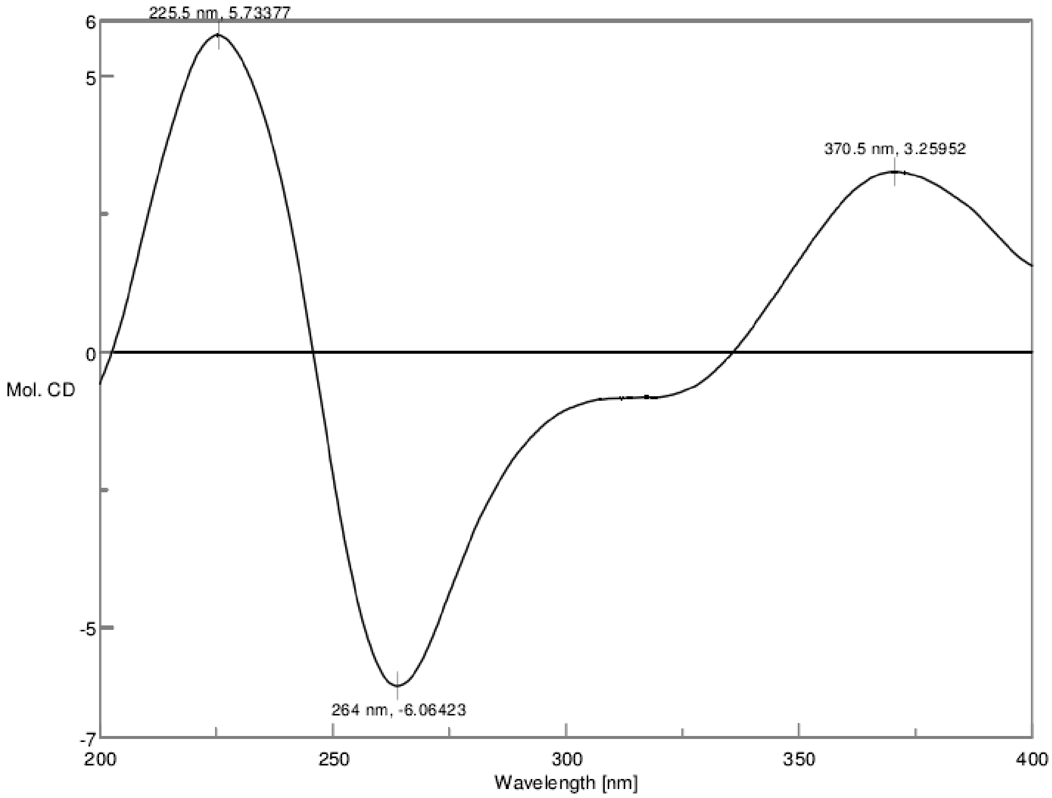

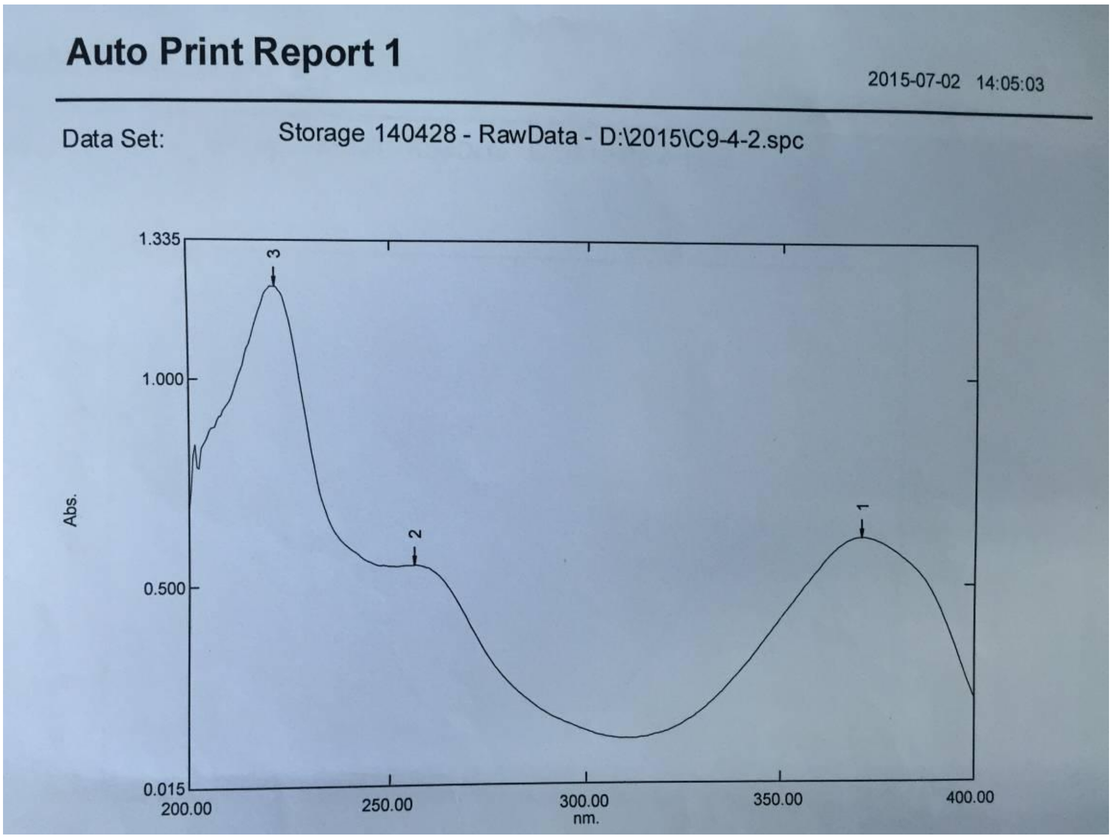

Fig. S20 ^{1}H NMR spectrum (600 MHz) of **4** in Methanol-d_4

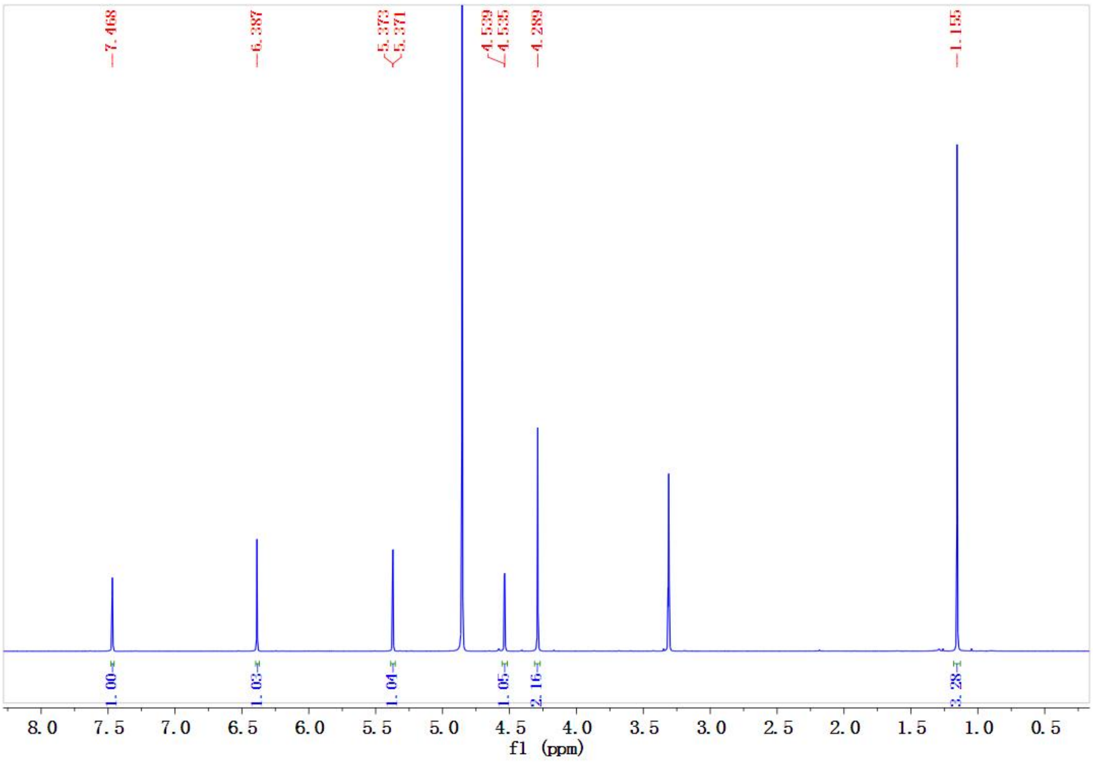

Fig. S21 ^{13}C NMR spectrum (150 MHz) of **4** in Methanol-d_4

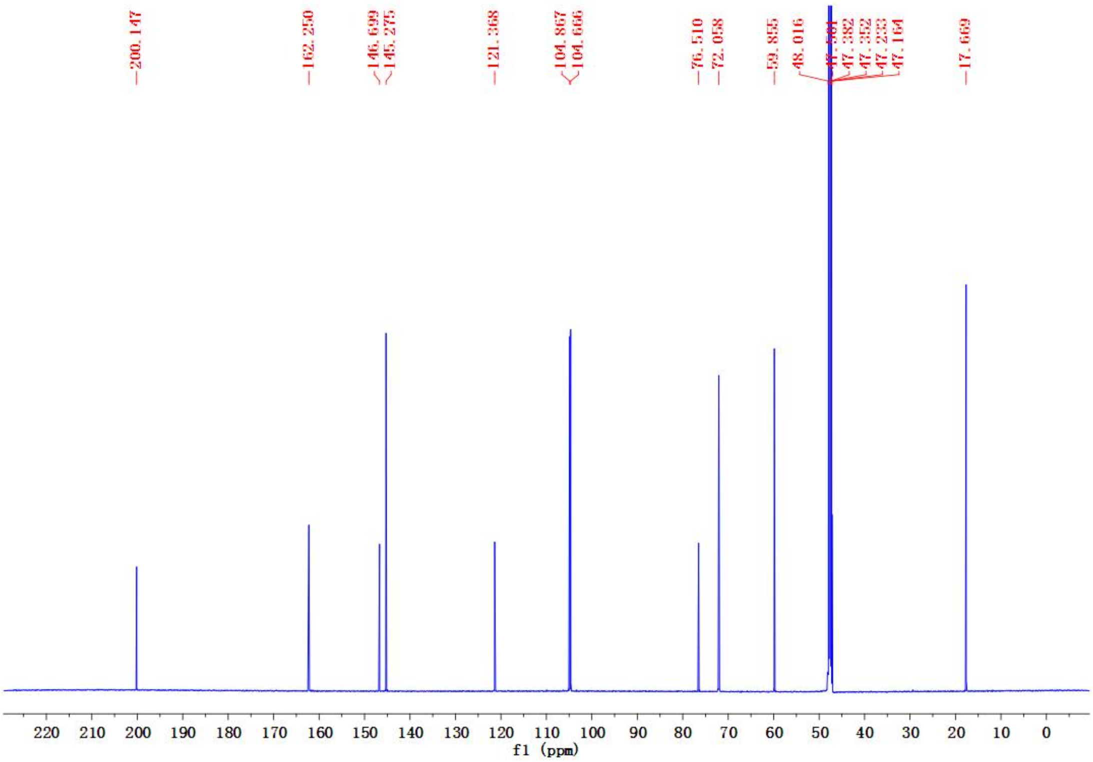

Fig. S22 HSQC spectrum of **4** in Methanol- d_4

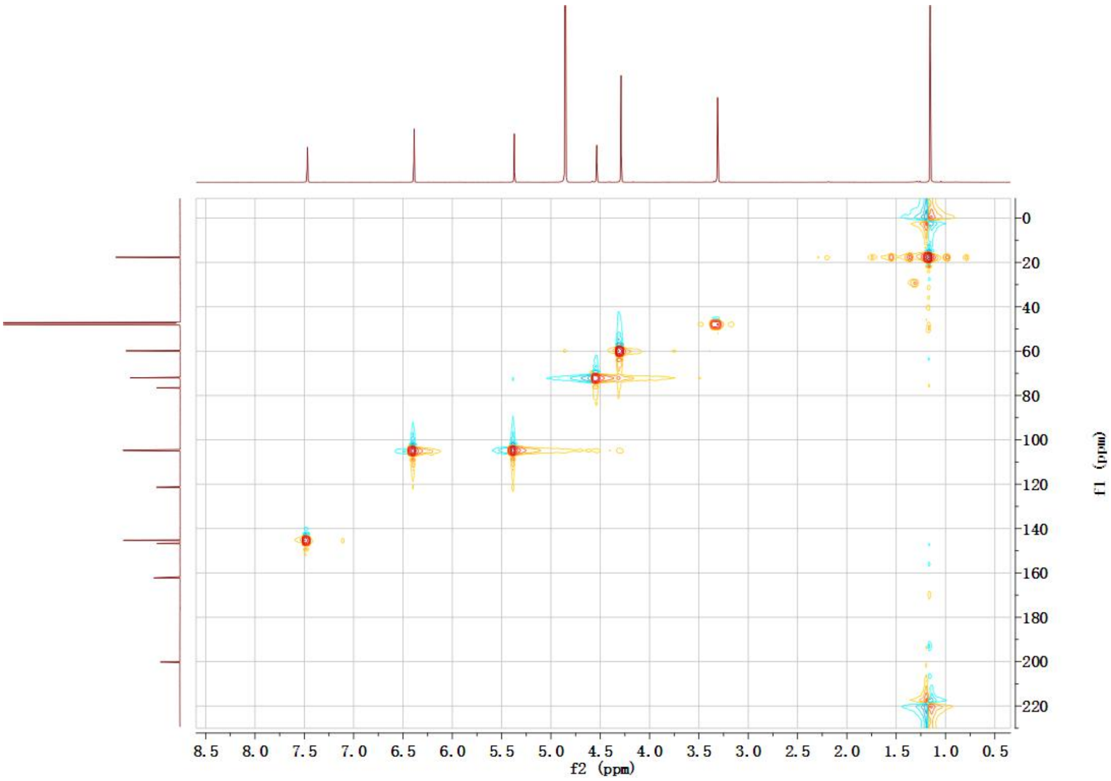

Fig. S23 HMBC spectrum of **4** in Methanol-d_4

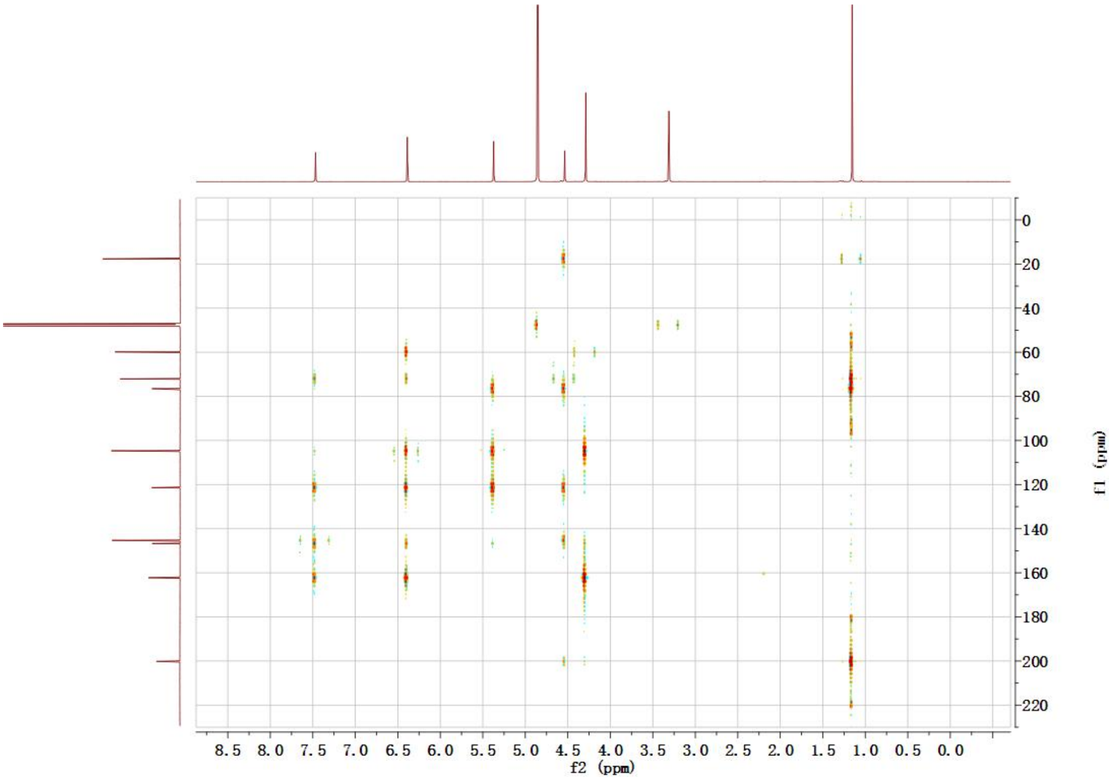

Fig. S24 HRESIMS spectrum of **4**

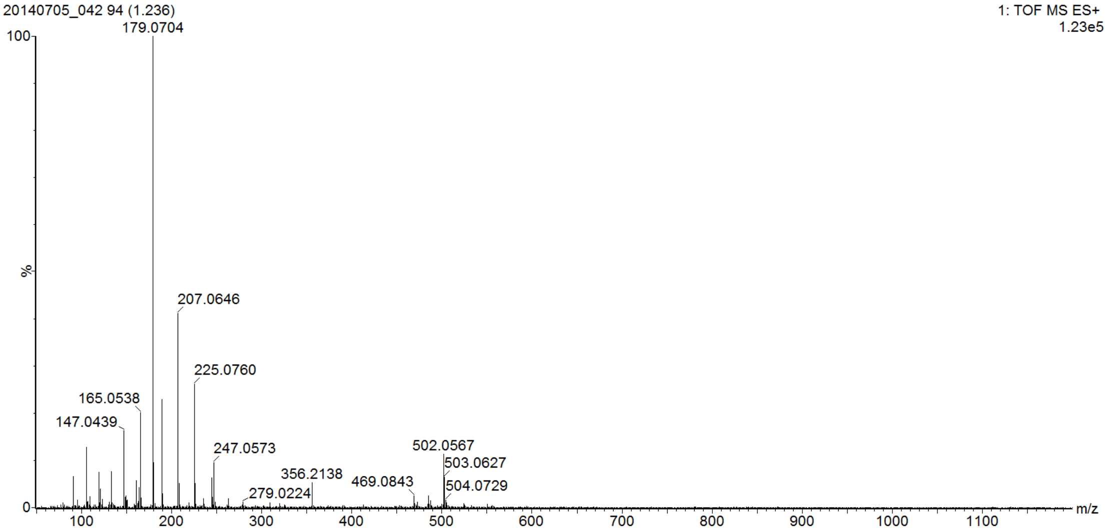

Fig. S25 IR (KBr disc) spectrum of 4

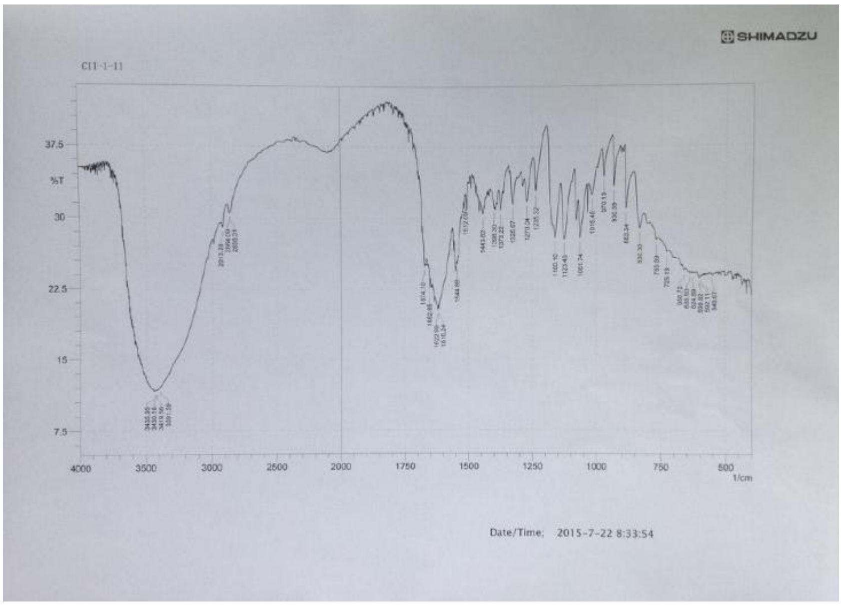

Fig. S26 CD and UV spectra of **4**

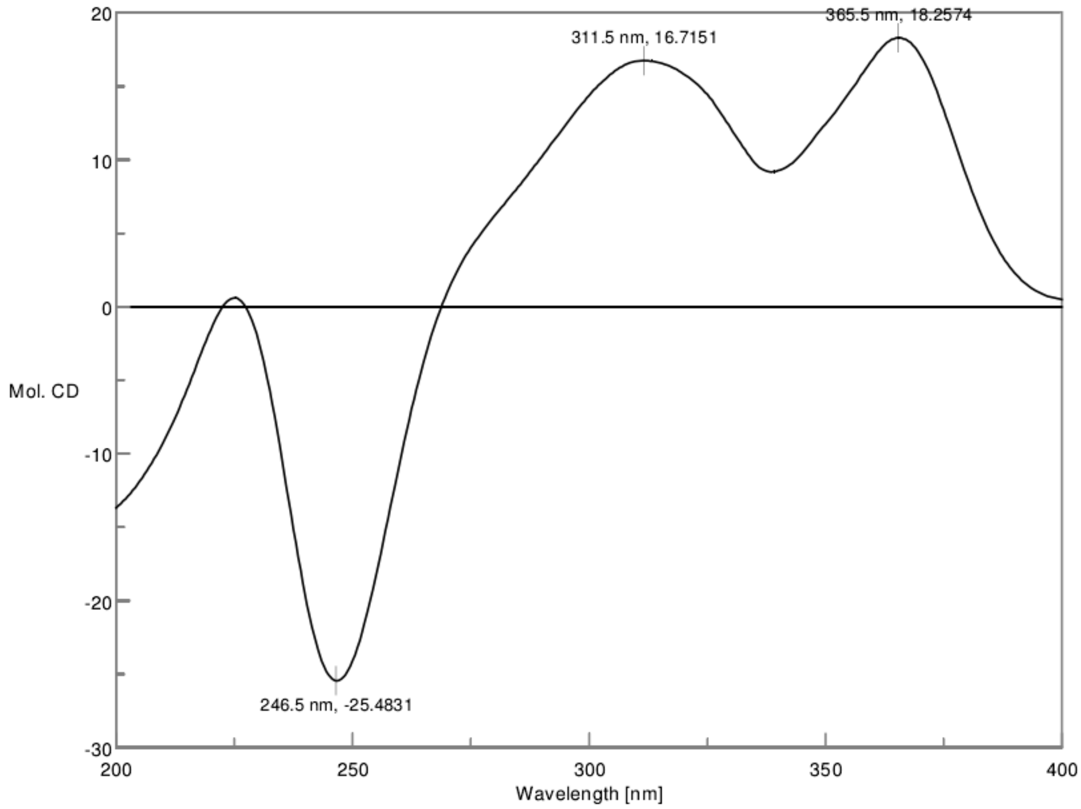

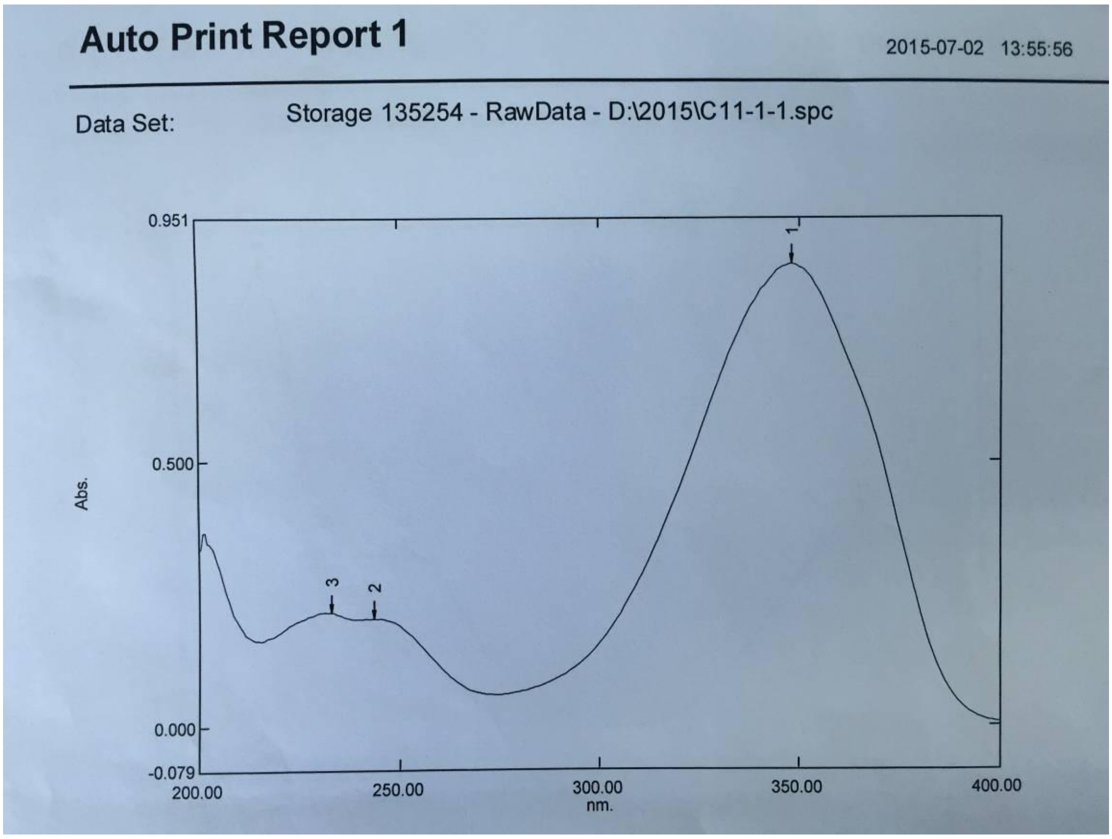

Supplement: Supplementary Information [file srep19350-s1.pdf]
